# Characterization of successive *ortho*-methyl oxidation of the priority pollutant 2,4-xylenol by a Rieske oxygenase system in *Pseudomonas putida* NCIMB 9866

**DOI:** 10.1128/aem.01293-26

**Published:** 2026-08-05

**Authors:** Haiqin Meng, Shun Pan, Zening Chai, Lixiang Zhou, Tianyu Zheng, Hanyu Zhang, He Fu, Lei Zou, Dongqi Liu, Jingcheng Dai, Dazhong Yan, Hong-Jun Chao

**Affiliations:** 1 School of Life Science and Technology, Wuhan Polytechnic University 74615, Wuhan, People's Republic of China; 2 Department of Biology, North Carolina Agricultural and Technical State University 3616, Greensboro, North Carolina, USA; 3 State Key Laboratory of Agricultural Microbiology, Huazhong Agricultural University 47895, Wuhan, People's Republic of China; 4 School of Environmental Science and Engineering, Huazhong University of Science and Technology 12443, Wuhan, People's Republic of China; 5 National Center of Technology Innovation for Synthetic Biology 658577 , Tianjin, People's Republic of China; Georgia Institute of Technology, Atlanta, Georgia, USA

**Keywords:** 2,4-xylenol, 4-hydroxy-3-methylbenzoate monooxygenase, methyl oxidation, Rieske oxygenase

## Abstract

The catabolism of the priority environmental pollutant 2,4-xylenol requires bacteria to oxidize its *ortho*-methyl group. Its exact biochemical mechanism is uncharacterized until now. In this study, we resolve key metabolic bottlenecks in *Pseudomonas putida* NCIMB 9866. We demonstrate that functionally redundant Rieske oxygenase systems ensure the robust *ortho*-methyl oxidation of 2,4-xylenol. On the *para*-methyl oxidation branch, PchA2 acts as the main aldehyde dehydrogenase. It shows significantly higher catalytic efficiency than the previously reported PchA. Additionally, we characterized PmmA1B1, a newly identified three-component Rieske oxygenase system responsible for *ortho*-methyl oxidation, which is supported by two redundant isoenzymes (PmmA2B2 and PmmA3B3), by recruiting the shared endogenous ferredoxin Orf05169. The PmmA1B1-Orf05169 system catalyzes the successive oxidation of the *ortho*-methyl group of 4-hydroxy-3-methylbenzoate to a carboxyl group, yielding the high-value pharmaceutical precursor 4-hydroxyisophthalate. Structural and kinetic data revealed that the active site architecture of PmmA1 enables efficient successive oxidation, distinguishing it from isoenzymes PmmA2B2 and PmmA3B3 that, when coupled with Orf05169, catalyze only incomplete oxidation. Overall, these results complete the 2,4-xylenol catabolic pathway by defining the enzymes required for both *para*- and *ortho*-methyl oxidation. They also reveal functional redundancy as an adaptive feature of microbial aromatic degradation and establish Rieske oxygenases as biocatalysts for converting methylated aromatic pollutants into value-added chemicals.

**IMPORTANCE:**

Methylated aromatics like 2,4-xylenol pose ongoing environmental risks and are primary targets for bioremediation. We know how bacteria break down many simple aromatics, but the precise ways that enzymes bypass the steric and electronic hurdles of hindered methyl groups remain unclear. In this study, we characterized a novel three-component Rieske oxygenase, PmmA1B1-Orf05169, capable of a rare successive oxidation that turns an *ortho*-methyl group directly into a carboxyl group. We also found parallel, redundant oxygenase pathways that protect the bacterium’s ability to degrade 2,4-xylenol during environmental stress or genetic loss. This discovery not only completes the metabolic map of 2,4-xylenol but also provides a robust biocatalytic tool for the green synthesis of 4-hydroxyisophthalate, a high-value pharmaceutical precursor.

## INTRODUCTION

**Fig 1:**
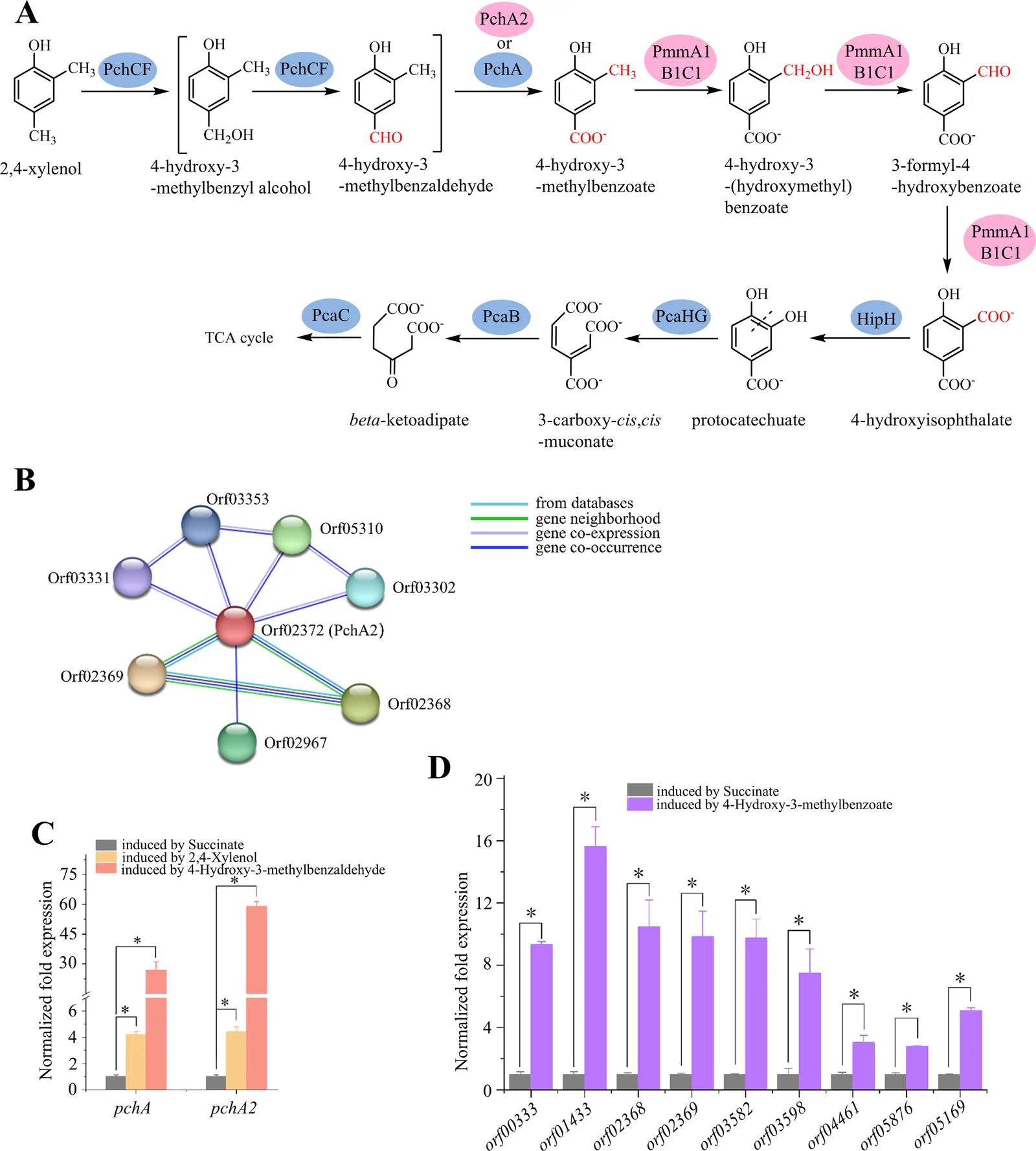
Discovery of enzymes that catalyze the conversion of 4-hydroxy-3-methylbenzoate and 4-hydroxy-3-methylbenzaldehyde (4H3MBH) in strain NCIMB 9866. (**A**) Proposed microbial metabolic pathway for 2,4-xylenol (14, 16, 18). Previously identified enzymes are indicated with blue ellipses. The enzyme marked with a pink ellipse represents the step identified in this study. (**B**) The potential 4-hydroxy-3-methylbenzoate monooxygenase identified through comparative genomic analysis of strain NCIMB 9866 using the STRING database. Transcriptional analyses of *p*-hydroxybenzaldehyde dehydrogenase- and 4-hydroxy-3-methylbenzoate monooxygenase-related genes in strain 9866 (**C and D**). The gene expression levels in each sample were calculated as fold expression ratios after normalization to 16S rRNA transcript levels. The expression level of the *orf03598* gene in strain 9866 induced with succinate was set to 1. *P < 0.05. Error bars represent one standard deviation (SD) of the mean values from three independent real-time quantitative PCR (RT-qPCR) experiments.

Methylated aromatic compounds represent a major class of industrial chemicals widely used in the production of pesticides, pharmaceuticals, and polymers (1–3). Their extensive usage and eventual release into the environment have caused persistent pollution and ecological toxicity, necessitating effective remediation strategies (4, 5). Among these, 2,4-xylenol (2,4-dimethylphenol) is listed as a priority pollutant by the U.S. Environmental Protection Agency (EPA) (6, 7). Understanding the microbial catabolism of such compounds is critical for predicting their environmental fate and developing biocatalytic systems for pollutant removal. Methyl substituents strongly influence a molecule’s reactivity and stability by shifting electron density and creating steric hindrance (8, 9). While the biodegradation of methyl groups typically proceeds via demethylation or oxidative transformation, the presence of *ortho*-substituents poses significant steric and electronic challenges, distinguishing their metabolic pathways from those of *para*- or *meta*-substituted isomers (10–12). Ring-methyl oxidation generally follows a stepwise sequence: methyl (-CH₃) → hydroxymethyl (-CH₂OH) → aldehyde (-CHO) → carboxyl (-COOH). Several enzymes catalyzing specific steps have been characterized in model organisms, such as *Comamonas testosteroni* T-2 (13) and *Pseudomonas putida* NCIMB 9866 (14). *P. putida* NCIMB 9866 was identified as early as the 1960s as a paradigm for the catabolism of methyl-substituted phenols (15). In this strain, the catabolism of 2,4-xylenol and *p*-cresol is primarily mediated by the genes on the pRA4000 plasmid, including the *pchACF* gene cluster (14). The oxidation of their *para*-methyl group is initiated by PchCF, a two-component flavin-dependent dehydrogenase. PchCF converts the methyl group into the corresponding alcohol, which is then oxidized to 4-hydroxy-3-methylbenzoate (4H3MBA) by *p*-hydroxybenzaldehyde dehydrogenase (PchA). Subsequently, the 4-hydroxyisophthalate hydroxylase encoded by the *hipH* gene converts the intermediate 4-hydroxyisophthalate into protocatechuate (16). While the *para*-oxidation branch is well-defined, microorganisms utilize diverse strategies to handle *ortho*-methyl groups of benzene rings. *Pseudomonas stutzeri* OX1 degrades *o*-cresol (2-methylphenol) via the adjacent hydroxylation of the benzene ring catalyzed by a phenol hydroxylase, yielding 3-methylcatechol (17). Despite such examples, the exact molecular mechanisms driving direct *ortho*-methyl oxidation have remained a missing link in the 2,4-xylenol pathway (Fig. 1A). This gap limits our comprehensive understanding of how microorganisms overcome the steric hindrance of *ortho*-methyl groups to achieve complete mineralization of 2,4-xylenol.

Furthermore, the intermediate product 4-hydroxyisophthalate (4HIPA) holds substantial industrial value. It serves as an organic scaffold for synthesizing non-steroidal anti-inflammatory drugs and anti-tumor agents with lower toxicity than aspirin (19–21). Commercial 4HIPA production currently relies on harsh chemical syntheses that generate toxic waste. Unlocking the enzymatic route for 4HIPA formation from pollutants like 2,4-xylenol offers a dual benefit: cleaning up environmental contamination while generating a valuable chemical precursor.

To fill these gaps, we elucidated the biochemical basis of *ortho*-methyl oxidation in *P. putida* NCIMB 9866. We report the discovery and characterization of a novel three-component Rieske oxygenase system, PmmA1B1-Orf05169. Unlike its isoenzymes (PmmA2B2-Orf05169 and PmmA3B3-Orf05169), which only catalyze the initial hydroxylation, PmmA1B1-Orf05169 facilitates the successive oxidation of the *ortho*-methyl group to a carboxyl group, yielding 4HIPA. By integrating kinetic analysis and structural modeling, this study highlights the functional divergence of Rieske oxygenases and their evolutionary ability to adapt to methylated contaminants. These findings not only complete the metabolic map of 2,4-xylenol but also provide a robust enzymatic tool for sustainable 4HIPA biosynthesis.

## RESULTS

### Genomic prediction of the gene encoding 4-hydroxy-3-methylbenzoate monooxygenase in *Pseudomonas putida* NCIMB 9866

In this study, we performed whole-genome sequencing and *de novo* assembly of *P. putida* NCIMB 9866. The complete genome consists of a single 6,340,806 base pair (bp) chromosome and an 85,027 bp plasmid, totaling 6,425,833 bp. The genes *pchCF* and *pchA* are all located on the pRA4000 plasmid, consistent with previous studies (22–24). However, the *hipH* gene (16) is annotated on the chromosome. Previous findings demonstrating that a *pchA* knockout mutant retains the ability to utilize 2,4-xylenol and *p*-cresol strongly suggest the presence of alternative *p*-hydroxybenzaldehyde dehydrogenases within the NCIMB 9866 genome (14). To identify these potential isoenzymes, a functional protein association network was constructed using the amino acid sequence of PchA and the complete proteome of NCIMB 9866 via the STRING database (25). This analysis revealed that Orf02372, subsequently designated as PchA2, shares 35.9% amino acid sequence identity with PchA. Furthermore, the network linked PchA2 to seven other uncharacterized proteins (Fig. 1B). Among these, Orf02368 and Orf02369 exhibited strong functional correlations with PchA2, suggesting they are likely involved in catalyzing the hydroxylation of 4H3MBA. Sequence analysis indicated that Orf02368 and Orf02369 share homology with components of the two-component enzyme phthalate 4,5-dioxygenase. Specifically, Orf02368 shares 21.7% identity with reductase component PhtB (GenBank accession no. EED68693) from *Comamonas testosteroni* KF1 (26), while Orf02369 has 27.3% identity with the oxygenase component PhtA (GenBank accession no. EED67076) from the same strain. Based on these homology results, Orf02368 and Orf02369 were designated as PmmB1 (4-hydroxy-3-methylbenzoate monooxygenase reductase component B1) and PmmA1 (4-hydroxy-3-methylbenzoate monooxygenase oxygenase component A1), respectively.

### The *pchA2* gene encodes an alternative dehydrogenase that catalyzes the oxidation of 4-hydroxy-3-methylbenzaldehyde

**Fig 2:**
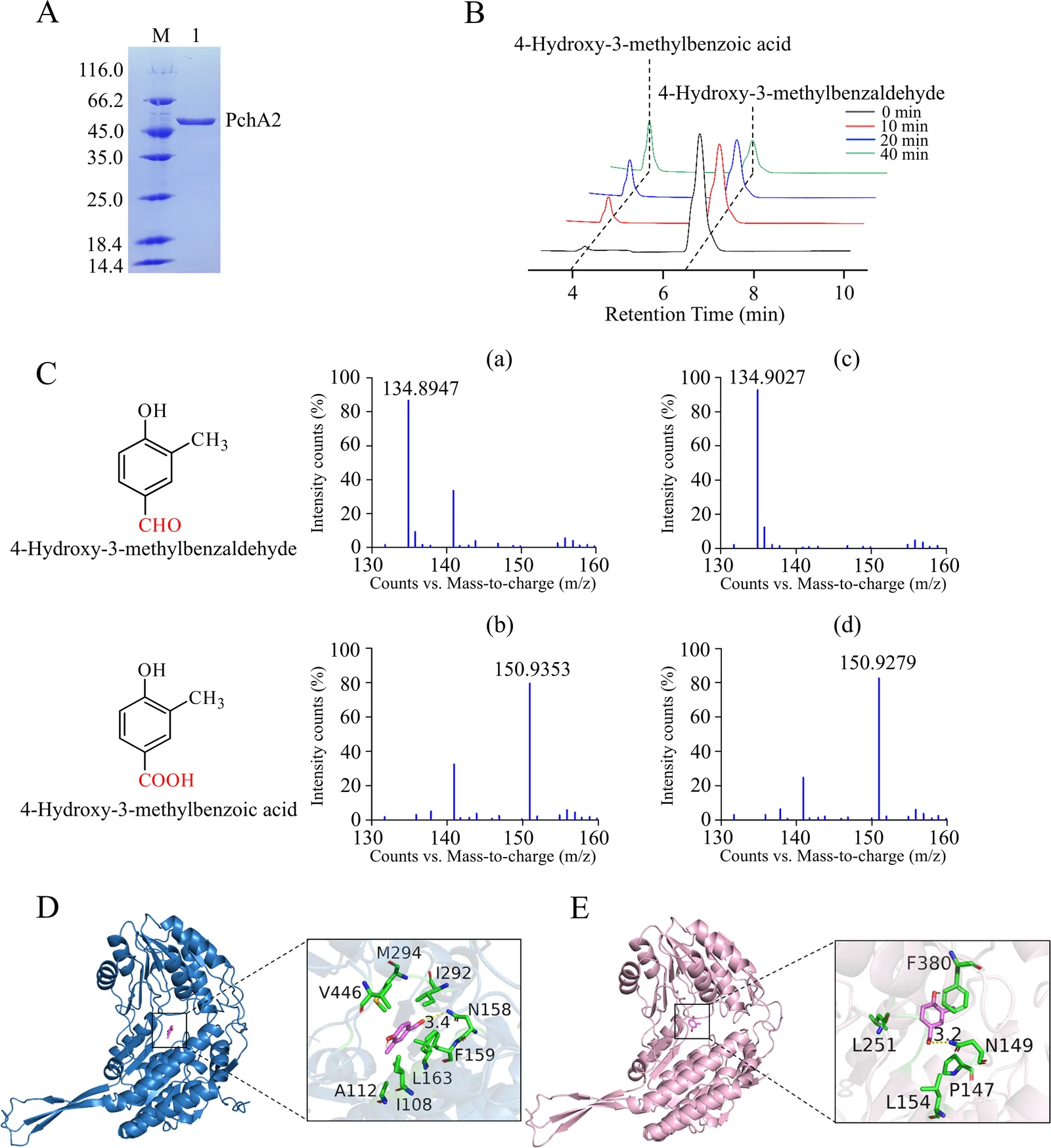
Purification, enzyme activity analysis, and overall structure of recombinant His_6_-PchA2. (**A**) SDS-PAGE analysis of purified recombinant His_6_-PchA2. (**B**) HPLC chromatograms showing biotransformation products of 4-hydroxy-3-methylbenzaldehyde catalyzed by recombinant His_6_-PchA2. Authentic 4-hydroxy-3-methylbenzaldehyde and authentic 4-hydroxy-3-methylbenzoic acid are shown in Fig. S1A. (**C**) LC-MS identification of the biotransformation products: mass spectra of 4-hydroxy-3-methylbenzaldehyde (a) and 4-hydroxy-3-methylbenzoic acid (b) detected during the biotransformation, along with authentic 4-hydroxy-3-methylbenzaldehyde (c) and authentic 4-hydroxy-3-methylbenzoic acid (d). The 4-hydroxy-3-methylbenzaldehyde substrate-binding pocket of PchA (**D**) and PchA2 (**E**) along with the surrounding side chains around the active site is shown in stick representation; the distance from the active site centers of PchA is given in Å. PchA is colored in sky blue, PchA2 in light pink, and 4-hydroxy-3-methylbenzaldehyde in pink. The structural models of PchA and PchA2 were predicted using AlphaFold 3 and aligned with the crystal structure of SALD from *Pseudomonas putida* G7 (PDB: 6QHN) (Fig. S1C).

Previous studies have reported that the *pchA* gene, which encodes *p*-hydroxybenzaldehyde dehydrogenase, catalyzes the oxidation of 4H3MBH during 2,4-xylenol catabolism (14). Transcriptional analysis revealed that *pchA2* expression was upregulated 4.4 ± 0.4-fold and 59 ± 2-fold when induced by 2,4-xylenol and 4-hydroxy-3-methylbenzaldehyde, respectively, compared to uninduced conditions (Fig. 1C). This induction pattern was similar to that observed for *pchA. In vitro* assays with the purified recombinant His_6_-PchA2 (Fig. 2A) demonstrated its ability to catalyze the dehydrogenation of 4-hydroxy-3-methylbenzaldehyde to 4H3MBA using either NADP^+^ or NAD^+^ as a co-substrate. Notably, the specific activity of PchA2 with NADP^+^ (16.9 ± 0.9 U mg^−1^) was significantly higher than its activity with NAD^+^ (0.379 ± 0.003 U mg^−1^). The identity of the biotransformation product was unequivocally confirmed as 4H3MBA through integrated high-performance liquid chromatography (HPLC) and liquid chromatography-mass spectrometry (LC-MS) analyses. The enzymatic product exhibited an HPLC retention time (3.92 min, Fig. 2B) identical to that of the authentic standard [Fig. S1A(b)]. Furthermore, LC-MS analysis in negative ion mode (Fig. 2C) confirmed that the *m*/*z* signals of the initial substrate [*m*/*z* 134.8947, Fig. 2C(a)] and the enzymatic product [*m*/*z* 150.9353, Fig. 2C(b)] matched the *m*/*z* values of the authentic 4-hydroxy-3-methylbenzaldehyde [*m*/*z* 134.9027, Fig. 2C(c)] and 4-hydroxy-3-methylbenzoic acid standards [*m*/*z* 150.9279, Fig. 2C(d)], respectively. Ultimately, PchA2 stoichiometrically converted 4-hydroxy-3-methylbenzaldehyde to 4-hydroxy-3-methylbenzoic acid (Fig. S1B). To evaluate the catalytic performance and substrate preferences of PchA and PchA2, steady-state kinetic assays were conducted (Table 1). While both enzymes can catalyze the oxidation of 4-hydroxy-3-methylbenzaldehyde to 4H3MBA, PchA2 exhibited a marked preference for this substrate. Its catalytic efficiency (*k*_cat_/*K_m_*) for 4-hydroxy-3-methylbenzaldehyde was over an order of magnitude higher than that of PchA. This stark difference underscores the highly efficient and specialized role of PchA2 in driving the oxidation process forward. The physiological role of PchA2 was further corroborated by *in vivo* phenotypic analysis of deletion mutants. As shown in Table S1, while the single deletion of *pchA2* did not abolish the strain’s ability to grow on 4H3MBH, the double mutant NCIMB 9866Δ*pchA*Δ*pchA2* entirely lost this capability. This indicates that despite the superior *in vitro* catalytic efficiency of PchA2, PchA and PchA2 function redundantly *in vivo*. Additionally, PchA2 exhibited a specific activity of 9.3 ± 0.9 U/mg toward vanillin. These results confirm that PchA2 is an aldehyde dehydrogenase with a strong substrate preference for 4-hydroxy-3-methylbenzaldehyde rather than that of vanillin.

**TABLE 1:** Apparent steady-state kinetic parameters of purified His_6_-PchA and His_6_-PchA2 toward 4-hydroxy-3-methylbenzaldehyde*^a^*

Substrate	Enzyme	Co-substrate	*K*_m_ (μM)	*k*_cat_ (s^−1^)	*k*_cat_/*K*_m_ (μM^−1^ s^−1^)
4-Hydroxy-3-methylbenzaldehyde	PchA*^b^*	NADP^+^	6 ± 1	4.3 ± 0.3	0.7 ± 0.1
		NAD^+^	21 ± 2	0.100 ± 0.002	0.0049 ± 0.0005
	PchA2	NADP^+^	1.8 ± 0.1	15 ± 1	9 ± 1
		NAD^+^	5.8 ± 0.4	0.360 ± 0.003	0.0621 ± 0.0009

^*a*^ The error represents one SD of the mean values from the biological replicate experiments (*n* ≥ 3). Kinetic parameters determined at fixed experimental concentrations of NADH and ambient O_2_.

^*b*^ Data for PchA are from reference 14.

To elucidate the structural basis for the differing catalytic efficiencies between PchA and PchA2, molecular docking and dynamics simulations were performed. Their 3D structures were first modeled using AlphaFold 3 (Fig. S1C), yielding predicted template modeling (pTM) scores of 0.96 for PchA and 0.97 for PchA2, which indicate high structural reliability. These predicted models closely resemble the crystal structure of salicylaldehyde dehydrogenase (SALD) from *Pseudomonas putida* G7 (PDB: 6QHN). Structural alignment of the alpha carbons against SALD yielded root mean square deviation (RMSD) values of 1.790 Å and 1.160 Å for PchA and PchA2, respectively. These metrics confirm that the overall folding topology is highly conserved. Docking results (Fig. 2D and E) showed that PchA binds 4-hydroxy-3-methylbenzaldehyde with a binding free energy (ΔG) of −5.741 kcal/mol. In this complex, a stable hydrogen bond forms between residue N158 and the ligand (3.4 Å), and the substrate is enveloped by a substantial hydrophobic pocket comprising seven residues (I108, A112, F159, L163, I292, M294, and V446) (Fig. 2D). Consistent with their conserved core folds, PchA2 binds the substrate with a comparable thermodynamic affinity (ΔG = −5.846 kcal/mol). Its binding configuration similarly relies on a distinct interaction network, forming a hydrogen bond with N149 (3.2 Å) and hydrophobic interactions with P147, L154, L251, and F380 (Fig. 2E).

Subsequent 100 ns molecular dynamics (MD) simulations further revealed significant differences in the dynamic behaviors of the two enzyme-substrate complexes (Fig. S2). While trajectory analyses confirmed that the substrate 4-hydroxy-3-methylbenzaldehyde remained bound in both enzymes throughout the simulation, the PchA-substrate complex exhibited notable RMSD fluctuations between 30 and 100 ns (Fig. S2A). This instability likely stems from its larger, smoother binding pocket, which lacks sufficient geometric constraints and allows the substrate to frequently adopt non-productive conformations. In contrast, the PchA2-substrate complex underwent an initial structural rearrangement during the first 60 ns before stabilizing into a rigid, highly favorable catalytic conformation (Fig. S2B). MM-PBSA calculations further supported this stabilization, yielding binding free energies (ΔG_MMPBSA_) of −31.67 ± 1.46 kJ/mol for PchA (0 ns–30 ns) and a much stronger −45.91 ± 0.42 kJ/mol for PchA2 (90 ns–100 ns), indicating thermodynamically favorable binding driven primarily by van der Waals interactions. Ultimately, these MD simulations demonstrate that the tighter pocket and optimized spatial constraints of PchA2 (such as the C284 substitution for F389) dynamically restrict the substrate into a stable near-attack conformation, thereby explaining its superior *in vitro* catalytic efficiency.

### PmmA1B1-Orf05169 catalyzes the successive oxidation of the methyl groups of 4-hydroxy-3-methylbenzoate to 4-hydroxyisophthalate

**Fig 3:**
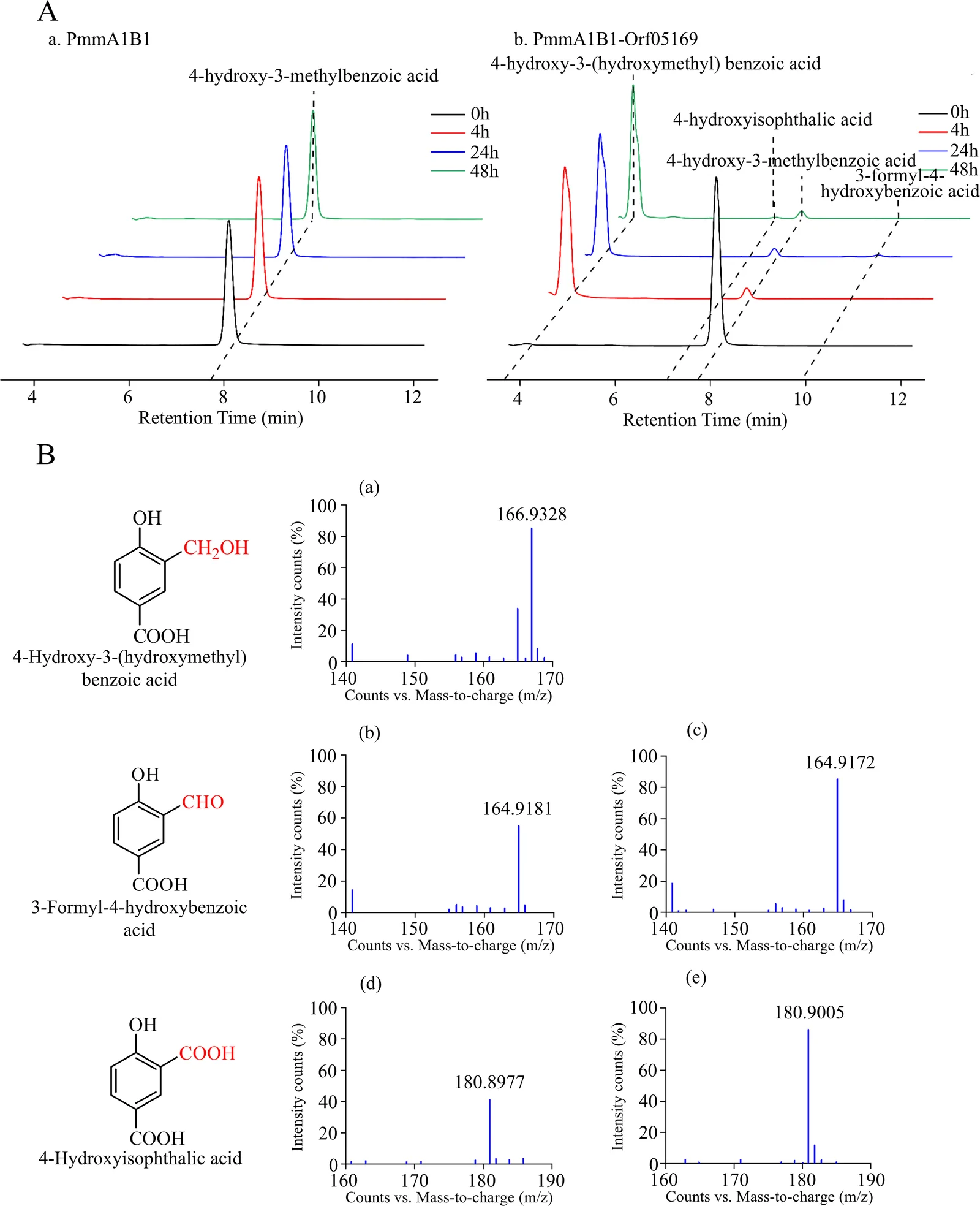
The enzyme activity analysis of recombinant His_6_-PmmA1B1 with ferredoxin. (**A**) HPLC chromatograms showing the biotransformation products of 4-hydroxy-3-methylbenzoic acid catalyzed by recombinant His_6_-PmmA1B1 with ferredoxin Orf05169. Results for His_6_-PmmA1B1 coupled with other ferredoxins are shown in Fig. S6. Authentic 4-hydroxy-3-methylbenzoic acid, authentic 3-formyl-4-hydroxybenzoic acid, and authentic 4-hydroxyisophthalic acid are shown in Fig. S6. (**B**) Identification of biotransformation products of 4-hydroxy-3-methylbenzoic acid by LC-MS. Mass spectra of 4-hydroxy-3-methylbenzoic acid (a), 4-hydroxy-3-(hydroxymethyl)benzoic acid (c), and 3-formyl-4-hydroxybenzoic acid (d) captured during the biotransformation. Mass spectra of authentic 4-hydroxy-3-methylbenzoic acid (b) and authentic 3-formyl-4-hydroxybenzoic acid (e).

Sequence analysis predicted that the *pmmA1B1* (*orf02369-02368*) genes encode an enzyme complex capable of catalyzing the hydroxylation of 4H3MBA (Fig. 1). To test this prediction, the components were heterologously expressed in *Escherichia coli* and functionally characterized (Fig. S3A). Initial *in vitro* assays with the purified recombinant His6-PmmA1B1 complex revealed no catalytic activity toward 4-hydroxy-3-methylbenzoic acid, even with the supplementation of various co-substrates, including NADH, NADPH, FAD, or FMN [Fig. S3B(c)]. However, the whole-cell recombinant strain *E. coli* BL21(DE3)/pET28a-*orf02368-02369* (*pmmB1A1*) successfully catalyzed the oxidation of 4-hydroxy-3-methylbenzoic acid [Fig. S3B(b)], yielding the sequential products 3-formyl-4-hydroxybenzoic acid and 4-hydroxyisophthalic acid. Bioinformatic annotation classifies PmmA1 and PmmB1 as Rieske superfamily proteins, with PmmA1 serving as the oxygenase component and PmmB1 as the reductase. Phylogenetic analysis further categorized PmmA1 as a Class III Rieske oxygenase (Fig. S4), a group that typically requires a distinct ferredoxin component for functional electron transfer (27). This suggests that the purified PmmA1B1 still requires an additional ferredoxin to achieve catalytic turnover. However, sequence analysis of the genomic region immediately surrounding the *pmmA1B1* locus did not reveal a co-transcribed ferredoxin gene. Further annotation of the *P. putida* NCIMB 9866 genome identified four distinct genes encoding [2Fe-2S] ferredoxin: *orf02496*, *orf05169*, *orf05199*, and *orf05413*. Additionally, a ferredoxin-encoding gene, *fdx*, was identified in the *E. coli* BL21(DE3) genome (28). To identify a compatible electron transfer partner, these five genes were cloned into the pETDuet-1 vector for heterologous expression (Fig. S5). *In vitro* biotransformation assays demonstrated that combining recombinant His_6_-PmmA1B1 with any of the five candidate ferredoxins restored the oxidation of 4-hydroxy-3-methylbenzoic acid (Fig. 3A and Fig. S6A through D). Notably, both Orf05169 and Orf05413 supported the highest specific enzymatic activity, with catalytic efficiencies that were statistically indistinguishable from one another (Table S2). Because the *pmmA1B1* cluster naturally lacks a ferredoxin gene, these results suggest that the oxygenase system has evolved to flexibly recruit multiple endogenous ferredoxins for electron transfer. For subsequent *in vitro* biochemical and structural characterization, Orf05169 was selected as a representative functional ferredoxin. Together, PmmA1, PmmB1, and Orf05169 constitute a functional three-component Rieske oxygenase system that catalyzes the methyl oxidation of 4H3MBA. Native-PAGE analysis revealed that while the theoretical monomeric mass of recombinant His_6_-PmmA1 is 40.9 kDa, the purified protein migrated primarily as a ~228 kDa complex under non-denaturing conditions (Fig. S5B). Consistent with homologous Rieske oxygenase components such as DdmC (29), which typically function as trimers, this apparent molecular mass indicates that PmmA1 adopts a fundamental trimeric unit that further oligomerizes into a stable hexamer in solution.

The biotransformation of 4-hydroxy-3-methylbenzoic acid by recombinant His_6_-PmmA1B1 coupled with Orf05169 was evaluated, and the catalytic products were identified by HPLC and LC-MS analyses (Fig. 3; Fig. S6). HPLC profiles (Fig. 3A) demonstrated the effective consumption of 4-hydroxy-3-methylbenzoic acid (retention time ~7.97 min), which was corroborated by LC-MS in negative ion mode (*m*/*z* 150.9642 matching the standard *m*/*z* 150.9678, Fig. S7A). Concurrently, a significant intermediate peak emerged at ~4.00 min with an *m*/*z* of 166.9328 (Fig. 3B); lacking a commercial standard, this was assigned as 4-hydroxy-3-(hydroxymethyl)benzoic acid. A subsequent intermediate was identified as 3-formyl-4-hydroxybenzoic acid by matching its retention time (~9.89 min) and *m*/*z* (164.9181) to the authentic standard (*m*/*z* 164.9172). Finally, the ultimate product of the reaction was identified as 4-hydroxyisophthalic acid based on its *m*/*z* 180.8977 (matching the standard 180.9005). To validate the final catalytic step, recombinant His_6_-PmmA1B1-Orf05169 was incubated with the intermediate 3-formyl-4-hydroxybenzoic acid (Fig. S7B). Dual HPLC [Fig. S7B(a)] and LC-MS (Fig. S7C) analyses demonstrated the consumption of 3-formyl-4-hydroxybenzoic acid (retention time ~9.71 min, *m*/*z* 164.9136) and the concomitant accumulation of 4-hydroxyisophthalic acid (retention time ~7.00 min, *m*/*z* 180.8992), with both analytes matching their respective authentic standards. Furthermore, quantitative HPLC analysis (Fig. S8) confirmed that the total molar amount of the detected sequential products (3-formyl-4-hydroxybenzoic acid and 4-hydroxyisophthalic acid) equaled the amount of 4-hydroxy-3-methylbenzoic acid consumed, demonstrating a complete mass balance for the transformation. Therefore, the His_6_-PmmA1B1-Orf05169 system possesses the remarkable ability to catalyze the successive oxidation of the 4H3MBA methyl group, sequentially forming 4H3HMBA and 3F4HBA, and ultimately yielding 4HIPA. Notably, over the course of this successive transformation (Fig. 3A; Fig. S6), the rapid depletion of 4-hydroxy-3-methylbenzoic acid and the marked concurrent accumulation of 4-hydroxy-3-(hydroxymethyl)benzoic acid at extended time points (e.g., 4 h, 8 h, 24 h, and 48 h) suggest that the initial hydroxylation of the methyl group occurs at a significantly faster rate than the subsequent oxidation steps. This accumulation pattern clearly indicates that the conversion of the hydroxylated intermediate [4-hydroxy-3-(hydroxymethyl)benzoic acid] constitutes the rate-limiting step in this sequential reaction pathway. Stoichiometric analysis further revealed that the successive oxidation catalyzed by PmmA1B1-Orf05169 proceeds with an overall NADH coupling efficiency of 86.2 ± 3.4%. Functionally, the resulting 4HIPA is subsequently targeted by 4-hydroxyisophthalate hydroxylase (encoded by the *hipH* gene) to produce protocatechuate (16), then enters the β-ketoadipate pathway for further catabolism. Finally, to assess substrate specificity, His6-PmmA1B1-Orf05169 was tested against a panel of structurally related aromatic compounds. It exhibited no monooxygenase activity toward the tested six xylenol isomers and the three cresol isomers (Table S3). In contrast, it displayed demethylation activity toward ring substrates containing methoxy groups, including 2-methoxyhydroquinone, vanillate, and guaiacol (Table S3).

### Strain NCIMB 9866 harbors multiple isozymes involved in 4-hydroxy-3-methylbenzoate oxidation

The Rieske oxygenase PmmA1B1-Orf05169, encoded by *orf02369-02368* and *orf05169*, catalyzes the hydroxylation of 4H3MBA (Fig. 3). To quantitatively assess the physiological significance, we generated a deletion mutant (NCIMB 9866Δ*orf02368-02369*) and monitored its growth dynamics. As shown in Fig. S9, the mutant exhibited robust growth on both 2,4-xylenol and 4H3MBA, with kinetics comparable to the wild-type strain NCIMB 9866. This sustained growth suggests a degree of metabolic redundancy, indicating that *orf02368-02369* is not the sole genetic locus capable of mediating 4H3MBA oxidation. To identify potential isoenzymes, we performed a genome-wide search using PmmA1B1 as a query, identifying six candidate systems (Table S4). Among them, Orf00334 and Orf05877 are not identical to PmmA1. Previous studies showed that the expression of the gene encoding 4-hydroxy-3-methylbenzoate monooxygenase is induced by 2,4-xylenol relative to succinate in strain NCIMB 9866 (30). Transcriptional analysis further supported their involvement; in cells grown on 4H3MBA, *orf02368* (*pmmB1*) and *orf02369* (*pmmA1*) were upregulated 10.5 ± 1.7-fold and 9.8 ± 1.6-fold, respectively, compared to succinate-grown controls (Fig. 1D). Notably, the ferredoxin-encoding gene *orf05169* also exhibited a 5.1 ± 0.2-fold increase in expression. Other candidate genes (*orf00333*, *orf01433*, *orf03582*, *orf03598*, *orf04461*, and *orf05876*) also exhibited significant induction, ranging from 3.0-fold to 16-fold. Subsequent heterologous expression and biochemical characterization confirmed that Orf01432-01433 (PmmA2B2) and Orf03582-03583 (PmmA3B3) with ferredoxin Orf05169 had the function of 4-hydroxy-3-methylbenzoate monooxygenase, which can catalyze the oxidation of 4-hydroxy-3-methylbenzoic acid to produce 4-hydroxy-3-(hydroxymethyl)benzoic acid (Fig. S10). Phylogenetic analysis (Fig. S4) confirmed that PmmA1, PmmA2, and PmmA3 belong to the Class III Rieske non-heme iron-dependent oxygenase family. Interestingly, while multiple endogenous ferredoxins can support these reactions, Orf05169 emerged as the efficient electron shuttle for all three oxygenase complexes (PmmA1B1, PmmA2B2, and PmmA3B3). This highlights Orf05169 as a versatile component within the catabolic network of strain 9866. Collectively, these findings demonstrate that the redundant enzymatic network provides a critical adaptive mechanism. This redundant enzyme system represents a critical adaptive mechanism that guarantees the continuity and robustness of 2,4-xylenol catabolism. By preventing pathway collapse due to gene loss or environmental fluctuations, this configuration ensures the metabolic continuity and robustness of 2,4-xylenol catabolism.

### Molecular docking with 4-hydroxy-3-methylbenzoate and its monooxygenases

**Fig 4:**
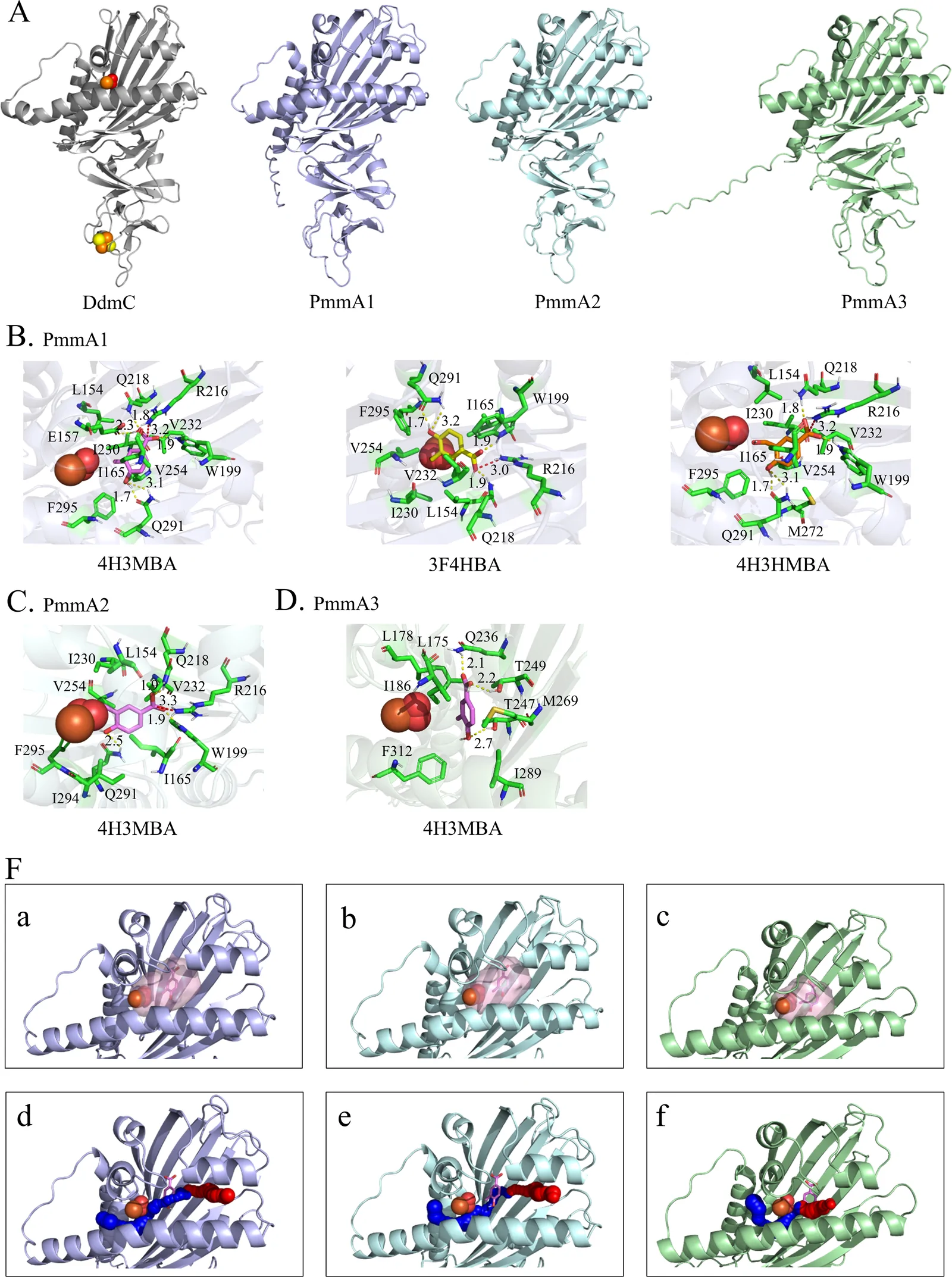
Overall structure of DdmC, PmmA1, PmmA2, and PmmA3. (**A**) Structural superposition of oxygenase DdmC (PDB: 3GL2) (light gray), PmmA1 (light blue), PmmA2 (pale cyan), and PmmA3 (pale green). The structures of PmmA1, PmmA2, and PmmA3 were predicted using AlphaFold 3 and aligned with the dicamba monooxygenase DdmC from *Stenotrophomonas maltophilia* DI-6 (PDB: 3GL2). The structures of PmmA1, PmmA2, and PmmA3 were predicted using AlphaFold 3 and aligned with the structures of PmmA1 (**B**), PmmA2 (**C**), and PmmA3 (**D**), and the residual side chains around the active site are shown in sticks. The substrates 4-hydroxy-3-methylbenzoate (4H3MBA), 3-formyl-4-hydroxybenzoate (3F4HBA), and 4-hydroxy-3-(hydroxymethyl)benzoate (4H3HMBA) pocket of PmmA1 and the residual side chain around the active site (B). The active site center of PmmA1 (**B**), PmmA2 (**C**), and PmmA3 (**D**) is shown, with distances given in Å. The salt bridges are represented by red dotted lines and the hydrogen bonds by yellow dotted lines. Iron atoms are represented in orange, sulfur atoms in yellow, and oxygen atoms in red. (**F**) Analysis of substrate binding pockets and tunnels in PmmA1, PmmA2, and PmmA3 using CavityPlus and CAVER Analyst 3.0, respectively. (a, b, and c) schematic diagrams of the binding pockets for PmmA1, PmmA2, and PmmA3 with 4-hydroxy-3-methylbenzoate. (d, e, and f) schematic diagrams of docking channels for PmmA1, PmmA2, and PmmA3 with 4-hydroxy-3-methylbenzoate. The binding pockets are shown in light pink, the substrate 4-hydroxy-3-methylbenzoate in violet, the channels for substrate entry in red, and the channels for product release in blue.

Unlike PmmA1B1, the isoenzymes PmmA2B2 and PmmA3B3 are incapable of catalyzing the successive oxidation of the 4H3MBA methyl group in the presence of ferredoxin, halting at the initial hydroxylation step. To explore the underlying structural and stereochemical factors that might dictate this observed *in vitro* functional divergence, structural modeling and molecular docking of the oxygenase components (PmmA1, PmmA2, and PmmA3) were performed as a comparative exploratory tool to investigate the mechanistic basis for the distinct characterized phenotypes. Three-dimensional structures were generated using AlphaFold 3, yielding high pTM scores for PmmA1 (0.92). These predicted models share high sequence identity (34.4%, 35.0%, and 35.8%, respectively) with the oxygenase component DdmC of dicamba O-demethylase from *Stenotrophomonas maltophilia* DI-6 (PDB: 3GL2). Structural superposition with the DdmC crystal structure (31) (Fig. 4A) produced low RMSD values (1.259 Å, 1.064 Å, and 1.087 Å) and high TM scores (0.933, 0.938, and 0.924), indicating highly conserved core folds across the three isoenzymes. Molecular docking of 4H3MBA revealed that all three oxygenase components exhibit favorable binding affinities within their catalytic pockets. The calculated minimum binding free energies (ΔG) were −6.226 kcal/mol for PmmA1, −6.730 kcal/mol for PmmA2, and −5.469 kcal/mol for PmmA3. These values align well with typical docking scores reported for specific binding of comparable small aromatic ligands in homologous Rieske dioxygenases (32). Given that empirical scoring functions like AutoDock Vina carry an inherent standard error of approximately 2.85 kcal/mol (33), minor numerical differences in these binding energies cannot serve as standalone evidence for binding affinity ranking. Therefore, subsequent analysis focused on their distinct spatial interaction networks. In PmmA1, the substrate is stabilized by five hydrogen bonds (E157 at 2.3 Å, W199 at 1.9 Å, Q218 at 1.8 Å, and two sites on Q291 at 1.7 Å and 3.1 Å), a salt bridge with R216 at 3.2 Å, and hydrophobic interactions involving L154, I165, I230, V232, V254, and F295 (Fig. 4B). Conversely, the substrate forms three hydrogen bonds (with W199 at 1.9 Å, Q218 at 1.9 Å, and Q291 at 2.5 Å) with PmmA2 (Fig. 4C), a salt bridge with R216 at 3.3 Å, and hydrophobic interactions with L154, I165, I230, V232, V254, I294, and F295. The substrate forms three hydrogen bonds with PmmA3 (with Q236 at 2.1 Å, T247 at 2.7 Å, and T249 at 2.2 Å) (Fig. 4D) and hydrophobic interactions with L175, L178, I186, M269, I289, and F312, but lacks salt bridges. Functionally, PmmA3 lacks salt bridges and forms relatively few hydrogen bonds—these involve uncharged polar amino acids. Despite a hydrophobic environment, the absence of synergistic electrostatic stabilization prevents PmmA3 from effectively supporting reaction intermediates or electron transfer, explaining its extremely limited catalytic efficiency. Although PmmA2 shares high structural similarity with PmmA1, its interaction network functions differently. PmmA2 heavily stabilizes the substrate’s ground state through a rigid network of hydrophobic interactions and hydrogen bonds. While this promotes initial substrate binding (reflected by a low *K_m_* value, Table 2), it simultaneously forces the substrate to overcome a high energy barrier to reach the transition state. This over-stabilization results in a very low apparent turnover rate (*k*_cat_ = 0.0018 ± 0.0002 s^−1^) and stalls the reaction, causing the intermediate 4H3HMBA to accumulate. In contrast, the hydrogen bond network of PmmA1 displays a “preorganized” architecture. Key hydrogen bonds (particularly with Q291 and E157), coupled with the electrostatic repulsion between the negative charge of E157 and the substrate’s carboxyl group, constrain the bound substrate in a high-energy state slightly deviated from its lowest-energy conformation. Based on these static docking models, we propose that this specific interaction network drives the ground state destabilization. Furthermore, this preorganized active site likely provides greater structural complementarity to the substrate’s transition state than to its ground state. The synergistic effect of ground-state destabilization and transition-state stabilization effectively lowers the activation energy barrier, enabling efficient and successive catalysis.

**TABLE 2:** Apparent steady-state kinetic parameters of the recombinant PmmA1B1, PmmA2B2, and PmmA3B3 systems coupled with ferredoxin Orf05169*^a^*

Substrate	Enzyme	*K*_m_ (μM)	*k*_cat_ (s^−1^)	*k*_cat_/*K*_m_ (μM^−1^ s^−1^)
4-Hydroxy-3-methylbenzoate	PmmA1B1-Orf05169	520 ± 110	0.013 ± 0.001	2.5 ± 0.6 × 10^−5^
	PmmA2B2-Orf05169	127 ± 2	0.0018 ± 0.0002	1.4 ± 0.2 × 10^−5^
	PmmA3B3-Orf05169	480 ± 70	0.0013 ± 0.0001	2.7 ± 0.5 × 10^−6^

^*a*^ The error represents one SD of the mean values from the biological replicate experiments (*n* ≥ 3). Kinetic parameters for the multi-component Rieske oxygenase systems determined at fixed experimental concentrations of NADH and ambient O_2_.

To further investigate this successive oxidation mechanism, docking was performed with the sequential intermediates 4H3HMBA and 3F4HBA (Table S5). Both intermediates exhibited favorable binding energies (−6.262 and −6.191 kcal/mol, respectively) comparable to the initial substrate (−6.226 kcal/mol). As shown in Fig. 4B, 4H3HMBA forms four stable hydrogen bonds (W199, Q218, and two with Q291), a salt bridge with R216 (3.2 Å), and specific hydrophobic interactions. Similarly, 3F4HBA forms four equivalent hydrogen bonds, a salt bridge with R216 (3.0 Å), and maintains a nearly identical hydrophobic interaction profile. Taken together, these results indicate that PmmA1 utilizes a highly conserved spatial interaction profile across all three substrates. The reliance on conserved key residues (W199, Q218, Q291, R216, L154, I165, I230, and V232) demonstrates that the PmmA1 active site structurally accommodates multiple reaction intermediates within a single pocket, requiring only minor conformational adjustments to facilitate successive, multi-step catalysis.

## DISCUSSION

In this study, 4-hydroxy-3-methylbenzoate monooxygenase PmmA1B1 coupled with Orf05169 from *P. putida* NCIMB 9866 exhibited continuous catalytic activity for the successive oxidation of the methyl group of 4H3MBA to a carboxyl group, producing 4HIPA (Fig. 3). This enzyme consists of the oxygenase component PmmA1, the reductase component PmmB1, and the recruited ferredoxin Orf05169. Notably, Orf05169 acts as a shared electron shuttle for multiple homologous oxygenase systems in strain 9866, including PmmA1B1, PmmA2B2, and PmmA3B3. El-Mansi and Hopper proposed that the enzyme responsible for oxidizing 4H3MBA evolved from vanillate demethylase (VanAB) (30). In *Pseudomonas* sp. strain ATCC 19151, vanillate demethylase (VanAB) converts vanillate into protocatechuate (34). Accordingly, VanAB (UniProt IDs P12609 and P12580, respectively) was used as the query sequence to search for potential homologs in the genome of strain NCIMB 9866, finding seven potential homologs (Table S4). Given the 69.0% sequence identity between PmmA1 and VanA, and *in vitro* assays showing that PmmA1B1-Orf05169 has comparable specific activities for vanillate (0.010 ± 0.001 U mg⁻¹) and 4H3MBA (0.018 ± 0.001 U mg⁻¹), PmmA1 might represent a native VanA with promiscuous activity. However, physiological and functional evidence supports its annotation as a 4H3MBA monooxygenase. The transcription of the *orf02369* (*pmmA1*) and *orf02369* (*pmmB1*) genes was induced by 4H3MBA (Fig. 1D), and the deletion mutant (NCIMB 9866 Δ*orf02368-02369*) showed no defect in vanillate utilization, indicating the presence of other dedicated VanA enzymes for native vanillate catabolism (Table S4 and Fig. S9B). Additionally, while PmmA1B1-Orf05169 retains baseline ability to perform single-step O-demethylation activity (Table S3), it catalyzes the multi-step, successive oxidation of the *ortho*-methyl group in 4H3MBA. Thus, while PmmA1 represents an example of functional divergence in microbial catabolism, its relatively low apparent specificity for 4H3MBA implies that its true, primary physiological substrate may yet be unidentified. It is probable that its native metabolic role involves a specific natural lignin derivative, and its ability to act as a successive oxidase of anthropogenic recalcitrant methylated pollutants represents a promiscuous adaptation of this ancient enzymatic pathway. Because lignin is a vital part of the land-based carbon pool, the microbial breakdown of these natural analogs is a key step in the carbon cycle (35, 36).

Regarding *para*-methyl oxidation, PchA2 was identified as the primary aldehyde dehydrogenase. Kinetic analyses (Table 1) show that PchA2 has a significantly lower *K_m_* and a catalytic efficiency (*k*_cat_/*K_m_*) approximately 12 times higher than PchA. Additionally, PchA2’s transcriptional level is twice that of PchA (Fig. 1C). Molecular docking and dynamics simulations suggest this difference relates to binding pocket volume. PchA’s larger pocket provides substrate flexibility but lacks effective orientational constraints, increasing the risk of non-productive binding. In contrast, PchA2’s moderately sized hydrophobic pocket imposes tighter spatial constraints, stabilizing productive conformations. These computational observations are consistent with the volume matching principle, suggesting that optimal catalytic efficiency may require the pocket volume to match that of the substrate. Excessively large or small pockets cause inadequate hydrophobic interactions or steric hindrance—with the latter being more detrimental—thereby reducing catalytic efficiency (37). Overall, these findings confirm PchA2’s primary role in oxidizing 4-hydroxy-3-methylbenzaldehyde in strain NCIMB 9866.

Functional redundancy among Rieske oxygenase systems contributes to the catabolism of 2,4-xylenol in strain NCIMB 9866. The deletion mutant Δ*orf02368-02369* exhibited growth on 4H3MBA comparable to the wild type (Fig. S9), indicating that compensatory isoenzymes (PmmA2B2-Orf05169 and PmmA3B3-Orf05169) can function *in vivo*. These isoenzymes exhibit functional differences *in vitro*: PmmA2B2-Orf05169 and PmmA3B3-Orf05169 catalyze only the initial hydroxylation step to yield the alcohol intermediate 4-hydroxy-3-(hydroxymethyl)benzoate, whereas PmmA1B1-Orf05169 uniquely catalyzes the successive multi-step oxidation directly to 4HIPA. This raises an apparent contradiction regarding how the deletion mutant (Δ*orf02368-02369*) can maintain robust growth on 4H3MBA if the remaining isoenzymes only perform the first step of the oxidation cascade. There are two probable, non-mutually exclusive explanations for this *in vivo* capability. First, as *Pseudomonas putida* harbors a vast arsenal of endogenous, broad-substrate alcohol and aldehyde dehydrogenases (38–40), it is highly likely that these background enzymes execute a metabolic hand-off. Once PmmA2B2 or PmmA3B3 overcome the initial activation barrier to form the alcohol, these native dehydrogenases rapidly oxidize it to the corresponding aldehyde and carboxylate, thereby rescuing pathway flux and enabling growth. Second, it is possible that the *in vitro* assay conditions do not fully capture their native catalytic potential; PmmA2B2 and PmmA3B3 might exhibit greater multi-step capacity *in vivo* when coupled with different physiological ferredoxins or specific cellular factors that are not present in the reconstituted system. Ultimately, the PmmA1B1 system demonstrates the specialized structural capacity for successive methyl oxidation. While the PmmA1B1-Orf05169 system demonstrates the unique capacity for successive methyl oxidation *in vitro*, its apparent steady-state kinetic parameters—the high *K_m_* and low *k*_cat_/*K_m_* (Table 2)—indicate a low intrinsic specificity for 4H3MBA. Because this system exhibits an efficient overall NADH coupling (86.2 ± 3.4%), this limited catalytic efficiency cannot be attributed to severe electron leakage or poor interaction with the recruited ferredoxin Orf05169. Instead, the high *K_m_* suggests that the oxygenase active site is not fully optimized for the high-affinity binding of 4H3MBA. Rather than viewing this as an artifact, these kinetic realities support the hypothesis that PmmA1 represents an evolutionarily promiscuous enzyme. Diverging from an ancestral vanillate demethylase (VanA), it has acquired the mechanistic ability to catalyze multi-step oxidation of anthropogenic methylated pollutants, even if it has not yet evolved maximal kinetic efficiency for these non-native substrates. Furthermore, while the sustained growth of the *orf02368-02369* deletion mutant hints at a robust metabolic redundancy network involving the isoenzymes PmmA2B2 and PmmA3B3, definitive proof of their collective physiological essentiality *in vivo* will require future investigations utilizing sequential multiple-knockout strains. Despite these inherent challenges of *in vitro* reconstitution, this division of labor expands the catalytic adaptability of strain NCIMB 9866.

The 4-hydroxy-3-methylbenzoate monooxygenase PmmA1B1-Orf05169 catalyzes the successive oxidation of the methyl group in 4H3MBA to produce 4H3HMBA and 3F4HBA, ultimately generating 4HIPA (Fig. 3), while the isoenzymes PmmA2B2-Orf05169 and PmmA3B3-Orf05169 catalyze the initial oxidation step to produce 4H3HMBA (Fig. S10). Thus, the significance of PmmA1B1-Orf05169 lies not in absolute physiological essentiality but in its remarkable and efficient multi-step catalytic architecture. The reliance on a shared ferredoxin (-Orf05169) by three different oxygenases mirrors other well-characterized multi-component systems, such as salicylate 5-hydroxylase and naphthalene dioxygenase (41). Such enzymatic “promiscuity” of electron transfer proteins is a common metabolic strategy to maximize catalytic flexibility while minimizing genetic load (42). PmmA2 has a large binding pocket (165.62 Å³) that synergizes with extensive hydrophobic interactions [Fig. 4F(b)] to rigidly immobilize the substrate. However, this excessively stabilizes the substrate’s ground state, thereby raising the transition state activation energy barrier. Without the preorganized, precise orientational constraints seen in PmmA1, the substrate exhibits high pocket flexibility in a non-productive conformation, resulting in a low productive binding probability (43) and a low turnover rate that stalls the reaction at the initial product stage. PmmA1 and PmmA3 possess moderately sized binding pockets [Fig. 4F(a and c)], but PmmA3 lacks the directional forces necessary to stabilize the high-energy transition state, leading to extremely low catalytic efficiency. Conversely, PmmA1 dynamically stabilizes the substrate in a reactive conformation through a preorganized active site network, heavily reliant on key residues Q291 and E157. Specifically, the electrostatic repulsion between E157 and the substrate’s carboxyl group decreases activation energy through two complementary mechanisms: destabilizing the substrate’s ground state while structural complementarity stabilizes the transition state, thereby enabling efficient, successive catalysis. Substrate entry into PmmA2’s tunnel is relatively smooth, whereas PmmA3’s longer, less flexible tunnel hinders catalytic initiation [Fig. 4F(d through f) and Table S6]. Although the product release tunnel of PmmA2 has a larger bottleneck radius and lower curvature, its average flow rate remains low, and its slightly longer length reduces permeability, which may cause product buildup and inhibition, further limiting turnover. The release tunnel of PmmA3 has the highest average flow rate and the shortest length, providing superior permeability that allows rapid and efficient product release (44–46). Its limited catalytic activity is mainly due to inefficient substrate-to-product conversion at the active site. For PmmA1, static analysis shows that its product release tunnel has low fluidity and a long length, but transient widening—driven by conformational fluctuations, residue flipping, and ring motions—facilitates product departure (47). Although transient widening can lead to brief product accumulation (48), the higher energy barrier of the chemical reaction step often remains the rate-limiting factor (49, 50). Slow release through a metastable tunnel intermediate (51) helps maintain the stability of the active site microenvironment—such as ordered water and charge distribution—reducing solvent disturbance of high-energy intermediates (52), which supports subsequent catalysis. Moreover, the spatially close active sites of Rieske oxygenases enable released products/intermediates to maintain high local concentrations, enhancing capture by adjacent monomers (53) and enabling multi-step reactions. Collectively, PmmA1’s binding pocket volume, channel fluidity, and the kinetic parameters of PmmA1B1-Orf05169 support its efficient, successive methyl group oxidation.

To analyze structural differences in the electron donor modules among the three 4-hydroxy-3-methylbenzoate monooxygenase systems, complete 3D models of the reductase components (PmmB1, PmmB2, and PmmB3) containing both the FAD cofactor and [2Fe-2S] cluster were constructed. AlphaFold 3 predictions of PmmB1 (pTM = 0.91), PmmB2 (pTM = 0.90), and PmmB3 (pTM = 0.91) showed the highest structural similarity with the phthalate dioxygenase reductase OphA1 from *Burkholderia cepacia* (UniProt: P33164), exhibiting sequence identities of 39.3%, 37.4%, and 41.2%, respectively. Structural alignment with the OphA1 crystal structure yielded low RMSD values (1.011 Å, 1.528 Å, and 1.351 Å) and high TM scores (0.756, 0.785, and 0.737) (Fig. S11A). The [2Fe-2S] cluster from OphA1 and the FAD cofactor from the homologous protein Hmp (UniProt: P39662) were incorporated into these structures for molecular docking, generating models with complete cofactor assemblies (Fig. S11B). The calculated minimum binding free energies of PmmB1, PmmB2, and PmmB3 with FAD were −10.34, −10.56, and −10.04 kcal/mol, respectively, indicating stable binding. However, pocket architectures varied significantly. Although PmmB2 exhibited the most negative binding free energy, its binding pocket was relatively small (898.5 Å³), accommodating primarily the N5 region near the isoalloxazine ring of FAD [Fig. S11C(b and e)]. This partial enclosure exposes other regions of FAD to the solvent and limits conformational constraints, resulting in locally constrained but globally unstable binding that may hinder electron transfer efficiency (54, 55). Similarly, PmmB3 possesses the smallest FAD binding pocket (855.75 Å³), with hydrogen bonding centered primarily on residue R73 [Fig. S11C(c and f)]. This localized binding pattern lacks uniform distribution, potentially limiting the conformational adaptability of FAD (56). In contrast, FAD in PmmB1 is fully enclosed within a larger binding pocket (1,606.75 Å³) and is surrounded by hydrophobic residues, including F218 and A220 [Fig. S11C(a and d)]. This hydrophobic environment reduces solvent interference and is supported by short hydrogen bonds that maintain conformational stability. The PmmB1 pocket balances spatial enclosure with sufficient internal volume, providing FAD with moderate conformational flexibility while maintaining stable binding (57). Predictions of the electron transfer pathways [Fig. S11C(g through i) and Table S7] further highlight these functional differences. The shortest edge distances between FAD-N5 and the [2Fe-2S] cluster are 11.0 Å for PmmB1, 14.4 Å for PmmB2, and 10.3 Å for PmmB3. Because the distance in PmmB2 exceeds the 14 Å threshold for direct electron tunneling, specific amino acid residues must act as relay sites for electron hopping (58–60). The predicted pathway for PmmB2 (FAD→Y216→Y287→[2Fe-2S]) has a total length of 33.8 Å and an edge weight of 29.59, indicating that electrons must pass through two tyrosine relays. This multi-step transfer increases the potential for energy loss. Conversely, the edge distances in PmmB1 and PmmB3 are below 14 Å, permitting direct electron tunneling. Electron transfer in both enzymes is predicted to follow a direct FAD→[2Fe-2S] pathway with lower edge weights (13.12 and 11.30, respectively), facilitating more efficient electron coupling. Collectively, the balanced FAD binding environment, shorter electron transfer pathway, and lower edge weight of PmmB1 support its capacity for efficient, successive catalysis.

### Conclusion

This study resolves a missing step in the bacterial catabolism of the priority pollutant 2,4-xylenol by detailing a novel three-component Rieske oxygenase system, PmmA1B1-Orf05169, in *Pseudomonas putida* NCIMB 9866. The newly identified isoenzymes (PmmA2B2-Orf05169 and PmmA3B3-Orf05169) stop at the initial hydroxylation. PmmA1B1-Orf05169, however, drives the successive oxidation of the sterically hindered *ortho*-methyl group of 4H3MBA directly to a carboxyl group. This activity provides a clear mechanistic model for *ortho*-methyl oxidation and generates 4HIPA, a valuable pharmaceutical precursor. Based on structural and kinetic data, PmmA1 achieves this multi-step efficiency via a preorganized active site network. This architecture destabilizes the substrate’s ground state and simultaneously provides structural complementarity to the transition state. An optimized electron transfer module in the reductase component further supports this process. Although the definitive endogenous physiological substrate remains to be fully elucidated, our results offer new perspectives on enzyme functional divergence and present a biocatalytic strategy to sustainably upcycle aromatic waste.

## MATERIALS AND METHODS

### Bacterial strains, plasmids, primers, chemicals, and culture media

Bacterial strains, plasmids, and primers used are listed in Tables S8 and S9. *Escherichia coli* was cultured aerobically in Luria-Bertani (LB) medium at 37°C with shaking (200 rpm) or on LB agar plates (1.5%, wt/vol). *Pseudomonas* strains were grown at 30°C in minimal medium supplemented with the specified growth substrates. Antibiotics were added as needed: ampicillin (100 μg/mL), kanamycin (50 μg/mL), or tetracycline (15 μg/mL). All reagents were purchased from Aladdin (Shanghai, China) or Sigma-Aldrich (St. Louis, MO, USA).

### Genome sequencing and assembly

*Pseudomonas putida* NCIMB 9866 was cultured and submitted to Shanghai Hanyu Bio-Tech Co., Ltd (Shanghai, China) for genomic DNA extraction and sequencing. Whole-genome sequencing was performed using the PacBio RS II sequencing platform, and *de novo* assembly was conducted using the PacBio SMRT Portal software. A total of 857 Mb of clean data were obtained after filtering adapters, short fragments, and low-quality reads, yielding an average read length of 5,335 bp. Gene prediction was performed using Glimmer gene-finding software (61).

### Phylogenetic analysis

The amino acid sequences of PmmA1, PmmA2, PmmA3, and VanA were used as queries for BLASTp searches against the UniProt database, retrieving eight homologous protein sequences from diverse bacterial genera and two manually curated monooxygenase sequences from *Pseudomonas* species. Initial multiple sequence alignments were performed using the MUSCLE algorithm in MEGA 12. To optimize these results, the preliminary alignments were refined using Clustal Omega, and the final sequence conservation was visualized using ESPript 3.0.

### Real-time quantitative PCR

Total RNA was extracted using the Monzol Reagent Pro (MonadBiotech, Wuhan, China) following the manufacturer’s protocol. RNA concentration and purity were assessed spectrophotometrically, and RNA integrity was confirmed by agarose gel electrophoresis. Subsequently, residual genomic DNA was removed, and the synthesis of first-strand cDNA was performed using the PrimeScript RT reagent Kit with gDNA Eraser (TaKaRa, Dalian, China) according to the manufacturer’s instructions. RT-qPCR analysis of gene transcription levels was conducted as previously described (14). The primer information is shown in Table S10. The amount of all target genes was normalized to that of 16S rRNA using the 2^-ΔΔ*C*^*^T^* method (62).

### Enzyme activity analysis and product identification

Recombinant proteins, including PchA2, PmmA1, PmmB1, and the various ferredoxins (Orf05169, Orf02496, Orf05199, Orf05413, and Fdx), were expressed in *E. coli* BL21(DE3) as His_6_-tagged fusion proteins and purified to homogeneity using Ni^2+^-NTA affinity chromatography, and further details are provided in the Supplementary Information. The cofactor content (iron, sulfur, and flavin) of the purified RO components was determined using colorimetric assays and UV-visible spectrophotometry to ensure the quality of enzyme preparations (Table S10). The activity assays for 4-hydroxy-3-methylbenzaldehyde dehydrogenase (14) and 4-hydroxy-3-methylbenzoate dehydrogenase (30) were conducted as previously described. The standard enzyme activity assay for the Rieske oxygenase system (PmmA1B1-Orf05169) was performed in a 1 mL reaction volume containing 50 mM HEPES-KOH buffer (pH 8.0), 200 μM of NADH or NADPH, 10 μM FAD, 50 μM Fe^2+^, and 300 μM L-ascorbate. To ensure that electron transfer was not rate-limiting during the assays, the reductase (His_6_-PmmB1) and ferredoxin (His_6_-Orf05169) components were provided in excess. For biotransformation and product identification assays, the multi-component reaction mixture contained high concentrations of the purified oxygenase (His_6_-PmmA1) at 50 μM–100 μM. The reductase (His_6_-PmmB1) and ferredoxin (His_6_-Orf05169) components were added at fixed, saturating concentrations to maintain at least a twofold and fourfold molar excess, respectively. The assay was initiated by the addition of 200 μM of either 4H3MBA or 3-formyl-4-hydroxybenzoate as the substrate. Reactions were incubated at 25°C and stopped at designated time points by adding an equal volume of ice-cold acetonitrile. The specific activities and kinetic parameters were properly normalized to the total protein concentration of the oxygenase component (PmmA1). One unit (U) of enzyme activity was defined as the amount of enzyme required to consume 1 μmol of substrate per minute under the standard assay conditions. The enzyme activity assays were conducted under steady-state conditions. To determine the steady-state kinetic parameters (*K_m_* and *k*_cat_), a final enzyme concentration of 0.01 μM was employed for His_6_-PchA2 kinetic assays, and 0.5 μM of the oxygenase component (His_6_-PmmA1) was used for the multi-component Rieske oxygenase systems. Initial reaction velocities were measured at a series of substrate concentrations: 0.5, 1, 2, 5, 10, 25, 50, and 100 μM for 4H3MBH, and 20, 50, 100, 200, 500, 1,000, 2,000, and 3,000 μM for 4H3MBA. All kinetic parameters were evaluated by fitting the experimental data to the Michaelis-Menten equation using non-linear regression in Origin 2024 (OriginLab, Northampton, MA). Each value represents the mean ± SD of at least three independent biological replicates.

Substrates and products withdrawn from the reaction were identified by HPLC or LC-MS, as adapted from previously reported protocols (63). Analytical separations were performed using an Elite EClassical 3100 HPLC system (Elite Analytical Instruments, Dalian, China) equipped with an Ultimate AQ-C18 column (4.6 × 250 mm, 5 μm, Welch Materials, Shanghai, China). The mobile phase consisted of solvent A (0.3% formic acid in water) and solvent B (acetonitrile) at a flow rate of 1.0 mL/min. Gradient elution was performed as follows: 0–5 min, 10% B; 5–25 min, 10%–75% B, with UV detection at 250 nm. LC-MS analysis was conducted using a QTRAP 4500 (AB Sciex, Framingham, MA, USA). Mass spectrometry parameters included a capillary voltage of 4,500 V, a drying gas temperature of 350°C, and a nebulizer pressure of 50 psi. Concentrations of 4H3MBA, 3F4HBA, and 4HIPA were calculated using standard calibration curves derived from commercial standards. 4H3HMBA was identified qualitatively via LC-MS but was excluded from absolute quantification due to the lack of a commercial standard.

### Molecular docking of protein to substrate

The 3D structures of the proteins used in this study were modeled using AlphaFold 3 (64) (https://alphafoldserver.com/). Models with the highest confidence scores (predicted local-distance difference test, pLDDT) were selected as receptors. Ligand structures were retrieved from the PubChem database (https://pubchem.ncbi.nlm.nih.gov/). Protein active sites were predicted by SWISS-MODEL. Molecular docking between the enzyme and substrate was performed using AutoDock Vina 1.2.7 software (35), with a 100 Å grid box centered on the predicted active site. Stable enzyme-substrate complexes were generated using the Lamarckian genetic algorithm, executing 100 independent runs with all other parameters set to default values. The docking results were visualized using PyMOL Molecular Graphics System (version 3.1.0, Schrödinger, LLC).

## Data Availability

The genomic data of strain NCIMB 9866 have been deposited in DDBJ/ENA/GenBank under the accession number JBRWOY000000000.

## SUPPLEMENTAL MATERIAL

Supplemental material aem.01293-26-s0001.pdf Supplemental methods, Fig. S1 to S11, and Tables S1 to S10.

ASM does not own the copyrights to Supplemental Material that may be linked to, or accessed through, an article. The authors have granted ASM a non-exclusive, world-wide license to publish the Supplemental Material files. Please contact the corresponding author directly for reuse.
