# Supplementary material for: Characterization of successive ortho-methyl oxidation of the priority pollutant 2,4-xylenol by a Rieske oxygenase system in Pseudomonas putida NCIMB 9866
Source: Appl Environ Microbiol. 2026 Aug 5;92(9):e01293-26. doi: 10.1128/aem.01293-26 (PMC13599478; doi:10.1128/aem.01293-26)
Supplement: Supplementary file 1 [file aem.01293-26-s0001.pdf]

Supporting Information

Characterization of Successive *ortho*-Methyl Oxidation of the Priority Pollutant 2,4-Xylenol by a Rieske Oxygenase System in *Pseudomonas putida* NCIMB 9866

Haiqin Meng[a], Shun Pan[a], Zening Chai[a], Lixiang Zhou[a], Tianyu Zheng[a], Hanyu Zhang[a], He Fu[b], Lei Zou[c], Dongqi Liu[d], Jingcheng Dai[a,e],Dazhong Yan[a,e], Hong-Jun Chao[a,e,*]

[a] School of Life Science and Technology, Wuhan Polytechnic University, Wuhan, 430023, P.R. China.

[b] Department of Biology, North Carolina Agricultural and Technical State University, Greensboro, NC 27411, United States.

[c] State Key Laboratory of Agricultural Microbiology, Huazhong Agricultural University, Wuhan, 430070, P.R. China.

[d] School of Environmental Science and Engineering, Huazhong University of Science and Technology, Wuhan, 430074, P.R. China.

[e]National Center of Technology Innovation for Synthetic Biology, Tianjin 300308, P.R. China.

[*]Corresponding author:

Hong-Jun Chao, School of Life Science and Technology, Wuhan Polytechnic University, No. 68 South Xuefu Road, Changqing Garden, Wuhan, P.R. China

E-mail: chaohongjun@whpu.edu.cn

This PDF file includes:

MATERIALS AND METHODS

FIGURES S1 to S11

TABLES S1 to S10

REFERENCES

MATERIALS AND METHODS

S1. Construction of strains and plasmids

DNA manipulation was performed according to previously established protocols (1). Genomic DNA of strain NCIMB 9866 was extracted using the TIANamp Bacteria DNA Kit (TIANGEN, Beijing, China) according to the manufacturer's instructions. The concentration and purity of the extracted DNA were determined using a NanoDrop One spectrophotometer (Shanghai, China), and DNA integrity was verified by agarose gel electrophoresis. The *orf02372* gene was PCR amplified using primers pET28a-2372E01 and pET28a-2372E02 from the genomic DNA of strain NCIMB 9866. Subsequently, the amplified gene was cloned into the NdeI/HindIII restriction sites of the pET28a(+) vector using the ClonExpress II One Step Cloning Kit (Vazyme, Nanjing, China) to construct plasmid pET-28a-*orf02372*. The *orf02368-02369* gene was amplified using primers pET28a-2368-69E01 and pET28a-2368-69E02, and subsequently cloned into the *Nde*I/*Hind*III sites of the pET28a(+) vector to construct pET-28a-*orf02368-02369*. The *orf00333-00334* gene was amplified using primers pET28a-333-34E01 and pET28a-333-34E02 to construct pET-28a-*orf00333-00334*. The *orf01432-01433* gene was amplified using primers pET28a-1432-33E01 and pET28a-1432-33E02 to construct pET-28a-*orf01432-01433*. The *orf03582-03583* gene was amplified using primers pET28a-3582-83E01 and pET28a-3582-83E02 to construct pET-28a-*orf03582-03583*. The *orf03598-03599* gene was amplified using primers pET28a-3598-99E01 and pET28a-3598-99E02 to construct pET-28a-*orf03598-03599*. The *orf04460-04461* gene was amplified using primers pET28a-4460-61E01 and pET28a-4460-61E02 to construct pET-28a-*orf04460-04461*. The same method was employed to construct vectors for the remaining genes.

The construction of plasmid pEX18Tc-*orf02368-02369* for gene knockout involved the fusion of PCR-amplified fragments flanking the *orf02368-02369* gene. Upstream and downstream fragments were generated using primers 9866-2368-69m01/9866-2368-69m02 and 9866-2368-69m03/9866-2368-69m04, respectively. These fragments were then cloned into the *Sma*I sites of the pEX18Tc plasmid using the ClonExpress II One Step Cloning Kit. The resulting plasmid was subsequently transformed into *E. coli* WM3064 and conjugated with strain NCIMB 9866, following a previously described protocol (2). Double-crossover recombinants of the mutant strain NCIMB 9866Δ*orf02368-02369* were screened on LB plates containing ampicillin and 15% (wt/vol) sucrose. For gene

complementation, plasmid pRK415-*orf02368-02369* was constructed by fusing the PCR product of *orf02368-02369*, amplified using primers orf02368-02369E3 and orf02368-02369E4, into the *Hind*III/*Eco*RI sites of the pRK415 plasmid. This plasmid was then transformed into *E. coli* WM3064 and mated with the mutant strain NCIMB 9866Δ*orf02368-02369* by conjugation, resulting in the generation of the complemented strain NCIMB 9866Δ*orf02368-02369*/pRK415-*orf02368-02369*.

S2. Gene expression and recombinant protein purification

To heterologously express the cloned genes, the recombinant plasmids (pET-28a(+) derivatives for *pchA2*, *orf02368-02369*, etc., and pETDuet-1 derivatives for *orf02368*, *orf02369*, *orf02496*, *orf05169*, *orf05199*, *orf05413*, and *fdx*) were transformed into *E. coli* BL21(DE3) cells (Novagen, Madison, WI, USA). For all recombinant proteins (PchA2, PmmA1, PmmB1, Orf05169, Orf02496, Orf05199, Orf05413, and Fdx), the cells were cultured aerobically at 37°C in LB medium supplemented with appropriate antibiotics (50 μg/mL ampicillin for pETDuet-1 or 50 μg/mL kanamycin for pET-28a(+)) until an OD_{600} of 0.4–0.6 was reached. Expression of the His_6-tagged protein was induced by adding 0.1 mM isopropyl-β-D-thiogalactopyranoside (IPTG), followed by incubation at 16 °C for 16 hours. Cells were harvested by centrifugation and disrupted by sonication. Purification of His_6-PchA2 was performed using Ni^{2+}-nitrilotriacetic acid agarose chromatography (Novagen), with elution achieved using 200 mM imidazole. To remove residual imidazole, the eluted proteins were dialyzed against 40 mM HEPES/KOH (pH 8.0) or 50 mM HEPES-NaOH (pH 8.0) at 4 °C for 24 hours using a Spectra/Por CE dialysis membrane with a 10,000 Da molecular weight cut-off (Spectrum Laboratories Inc., Shanghai, China). The purity and expected molecular weights of all purified proteins were rigorously assessed by sodium dodecyl sulfate-polyacrylamide gel electrophoresis (SDS-PAGE). Purified proteins were stored in 50% glycerol at -80 °C pending further enzymatic analysis. Protein concentrations were quantified using a BCA Protein Assay Kit (Sangon, Shanghai, China) following the manufacturer's protocol.

S3. Tricine-SDS-PAGE and Native-PAGE Analysis

The purified ferredoxin was assessed using Tricine-SDS-PAGE (3). The gel system consisted of a 16%

separating gel and a 4% stacking gel, prepared using an acrylamide-bisacrylamide stock (49.5% T, 3% C). Protein samples were mixed with a reducing buffer and incubated at 37 °C for 15 min without boiling. Electrophoresis was performed in an ice-water bath, initiated at 30 V until the dye front entered the separating gel, and then increased to 100–110 V. Following electrophoresis, the gels were fixed in 50% methanol and 10% acetic acid, stained with 0.025% Coomassie blue G-250, and subsequently destained in 10% acetic acid.

To determine the native oligomeric state of the purified Rieske non-heme iron monooxygenase Orf02369 (PmmA1), Native-PAGE was performed using an 8% resolving gel and a 4% stacking gel. Purified protein samples were mixed with a 4× native loading buffer (5 mL glycerol, 2 mL 0.5 M Tris-HCl [pH 6.8], 5 mg bromophenol blue, adjusted to 10 mL with deionized water) in the absence of SDS and β-mercaptoethanol. The samples were incubated on ice for 5 min without heat denaturation and centrifuged at 4 °C and 12,000 rpm for 10 min before loading. Electrophoresis was conducted at 4 °C using an SDS-free running buffer (14.4 g glycine and 5.05 g Tris, adjusted to 1 L with deionized water). The gel was run at 80 V for 30 min, followed by 120 V for 90 min. Following electrophoresis, the gel was stained with Coomassie Brilliant Blue R-250 (2.5 g dissolved in 450 mL methanol, 100 mL acetic acid, and deionized water to 1 L) and subsequently destained (450 mL methanol and 100 mL acetic acid, adjusted to 1 L with deionized water). The apparent molecular weight and oligomeric state were estimated by comparing the migration distance of the target protein to a high molecular weight native protein marker (BL1335A, Biosharp, Beijing, China).

S4. Determination of Cofactor Content

The iron content of purified PmmA1, PmmB1, and ferredoxins was determined using the ferrozine assay as previously described (4, 5). Acid-labile sulfur was quantified using the semi-micro method reported by Beinert (6). The flavin (FAD) content was determined by UV-visible absorption spectroscopy using an extinction coefficient of $\varepsilon_{450} = 11.3\ mM^{-1}\ cm^{-1}$ (7, 8). All measurements were performed in triplicate to calculate the molar ratio of cofactors per protein monomer.

S5. NADH Coupling Efficiency Assay

To determine the coupling efficiency of the PmmA1B1-Orf05169 system, the stoichiometry between

NADH oxidation and product formation was quantified. The NADH consumption assay was adapted from a previously published method (9) with minor modifications. Assays were conducted in 1 mL reaction volumes containing 50 mM HEPES-NaOH buffer (pH 8.0), 0.5 mM 4-hydroxy-3-methylbenzoate (4H3MBA), and a purified enzyme mixture of oxygenase (His_6-PmmA1) (50–100 µM), reductase (His_6-PmmB1), and ferredoxin (His_6-Orf05169) components at a molar ratio of 1:2:4 to ensure the oxygenase component was rate-limiting. The reactions were initiated by the addition of 2.0 mM NADH and incubated at 30 °C. The quantitative analysis of NADH was performed on an Elite EClassical 3100 HPLC system (Elite Analytical Instruments, Dalian, China) equipped with an Ultimate® AQ-C18 column (5 µm, 250 mm × 4.6 mm; Welch Materials, Shanghai, China). The column temperature was set at 20 °C with an injection volume of 10 µL and a flow rate of 1.0 mL/min. The mobile phase consisted of 50 mM ammonium acetate (solvent A) and 100% acetonitrile (solvent B). A gradient elution program was applied: 0 to 12 min, 0 to 10% B; 12 to 17 min, 10 to 0% B; and 17 to 20 min, 0% B. Dual-wavelength UV detection was utilized, with NADH strictly quantified based on its characteristic absorbance at 340 nm, while 260 nm served as a supplementary qualitative control. The concentrations of the aromatic substrate and downstream products were quantified via HPLC as described in the main text.

Because an authentic standard for the initial intermediate 4H3HMBA is commercially unavailable, its concentration was deduced mathematically via mass balance:

$$\Delta[4H3HMBA] = \Delta[4H3MBA]_{consumed} - \Delta[3F4HBA] - \Delta[4HIPA]$$

The apparent NADH coupling efficiency was then calculated as the ratio of the theoretical NADH equivalents required for all formed products to the actual NADH consumed during the initial linear phase of the reaction:

$$\text{Coupling Efficiency (\%)} = \frac{\Delta[4H3MBA]_{consumed} + \Delta[3F4HBA] + 2\times\Delta[4HIPA]}{\Delta[NADH]_{consumed}}$$

Where Δ[4H3MBA], Δ[3F4HBA], and Δ[4HIPA] represent the molar concentrations of the respective products formed, and Δ[NADH] represents the molar concentration of NADH consumed over the same time period.

S6. Molecular dynamics simulations

To evaluate the dynamic stability of the enzyme-substrate complexes, 100 ns molecular dynamics (MD) simulations were performed using GROMACS 2022.2 (10). The protein structures were parameterized using the Amber14SB force field (11). For the ligand (4-hydroxy-3-methylbenzaldehyde), AM1-BCC charges were generated using the antechamber module (12), and GAFF2 atom types were assigned before conversion to GROMACS topologies via ACPYPE. Each complex was solvated in a truncated dodecahedron box with TIP3P water molecules, and the system was neutralized with 0.15 M NaCl. Following energy minimization, the systems were sequentially equilibrated under NVT and NPT ensembles for 200 ps each at 298 K. The production MD simulations were run for 100 ns under NPT conditions with a 2-fs integration step. Long-range electrostatic interactions were treated using the Particle Mesh Ewald (PME) method, and hydrogen bonds were constrained using the LINCS algorithm. Trajectory analyses (RMSD, distance, and buried solvent-accessible surface area) were performed using GROMACS built-in tools, and the binding free energies were calculated using the gmx_MMPBSA tool (13).

S7. Analysis of Substrate-Binding Pocket and Channel

The obtained protein-substrate docking complexes were then analyzed using the CavityPlus platform web server (http://pkumdl.cn:8000/cavityplus/index.php#/) to predict the location of the substrate-binding pocket and calculate its volume, thereby evaluating the structural accommodation capacity of the different oxygenases. CAVER (14) was employed to predict tunnels in the complexes formed by each oxygenase with the substrate 4H3MBA. This analysis identified the potential pathways for substrate entry into the active site from the bulk solution and for product release after the reaction. By analyzing structural parameters of the channels, such as average throughput (Avg_throughput) and average length (Avg_L), the conformational characteristics of the substrate entry and product release channels were evaluated to assess their potential impact on catalytic turnover.

S8. Analysis of Electron Transfer Pathways

To investigate structural differences in the electron donor modules among the three oxygenase systems, structural modeling and electron transfer pathway prediction were performed for the reductases PmmB1, PmmB2, and PmmB3 within these systems. First, the functional domains of the reductase

components were annotated using InterProScan (15) (https://www.ebi.ac.uk/interpro/search/sequence/), which confirmed the presence of canonical FAD-binding domains and [2Fe-2S]-binding domains—key structural features for their electron transfer function. Using the AlphaFill platform (16), [2Fe-2S] clusters were extracted from resolved homologous protein structures and modeled into the target reductase structures according to their native metal coordination patterns. Furthermore, given the absence of standardized 3D coordinates for FAD molecules in the PubChem database, FAD coordinates were extracted from structural templates that natively contain FAD ligands. These FAD molecules were then incorporated into the predicted reductase structures via molecular docking, enabling the construction of 3D structural models with complete cofactor assemblies. Subsequently, CavityPlus (17) was employed to predict the FAD-binding pockets in the integrated reductase structures—providing insights into the spatial architecture that accommodates the FAD cofactor. Finally, using eMap (18) (https://emap.bu.edu/), the shortest electron transfer pathways from the FAD to the [2Fe-2S] cluster were predicted for each model. This analysis also identified key residues that may act as relays during electron hopping, providing a structural basis for evaluating differences in electron transfer efficiency and structural organization across the three oxygenase systems.

FIGURES

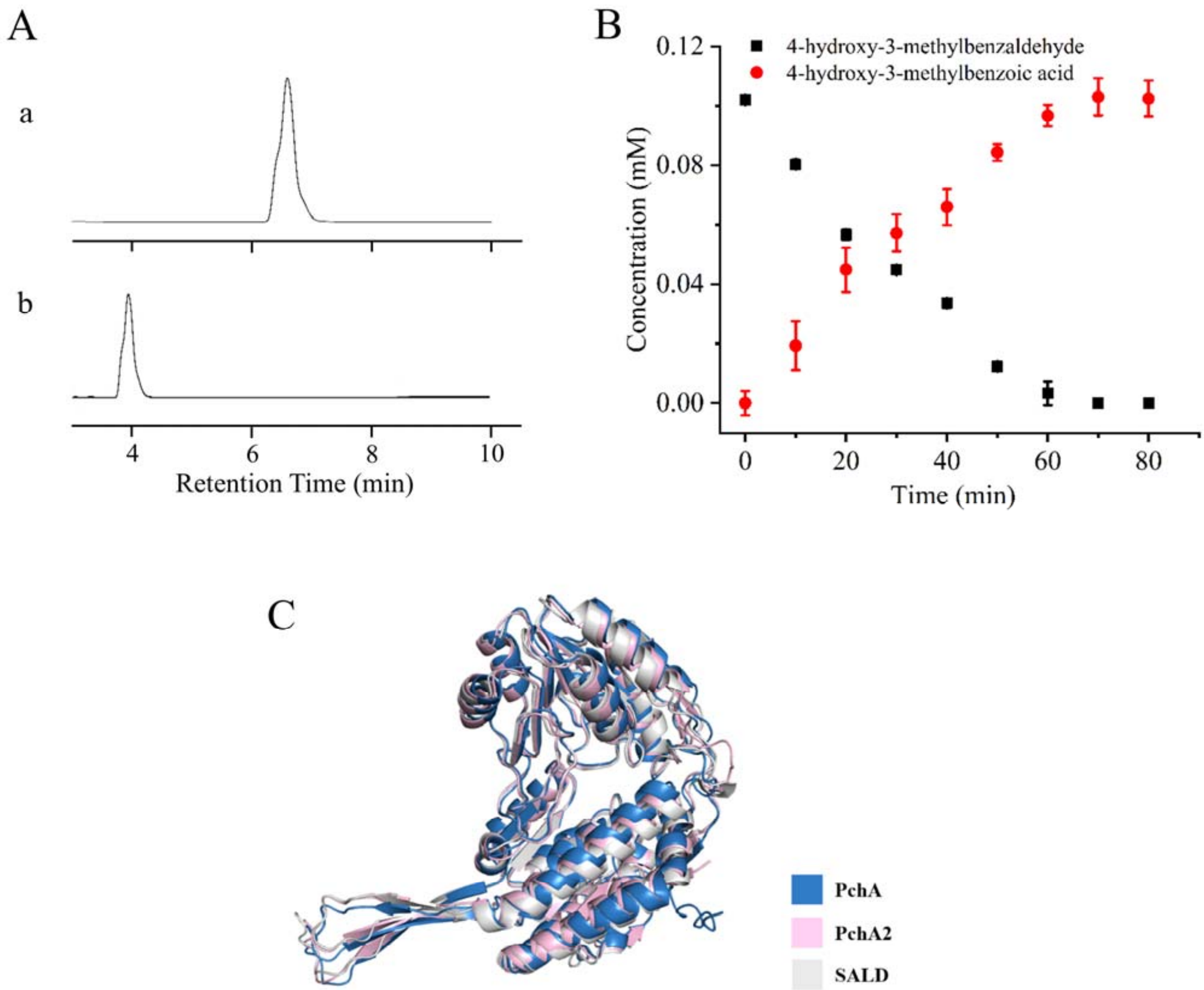

Figure S1 (A) HPLC chromatograms of the authentic 4-hydroxy-3-methylbenzaldehyde (a) and 4-hydroxy-3-methylbenzoic acid (b). (B) Time course of the oxidation of 4-hydroxy-3-methylbenzaldehyde catalyzed by purified His_6-PchA2. The consumption of 4-hydroxy-3-methylbenzaldehyde and the accumulation of 4-hydroxy-3-methylbenzoic acid were quantified via HPLC analysis. The error bars indicate one standard deviation (SD) of the mean values from three independent experiments. (C) Structural superposition of PchA, PchA2, and salicylaldehyde dehydrogenase (SALD). Structural models of PchA and PchA2 predicted by AlphaFold 3 were aligned with the crystal structure of SALD from *Pseudomonas putida* G7 (PDB: 6QHN). PchA is colored in sky blue, PchA2 in light pink, and SALD in light grey.

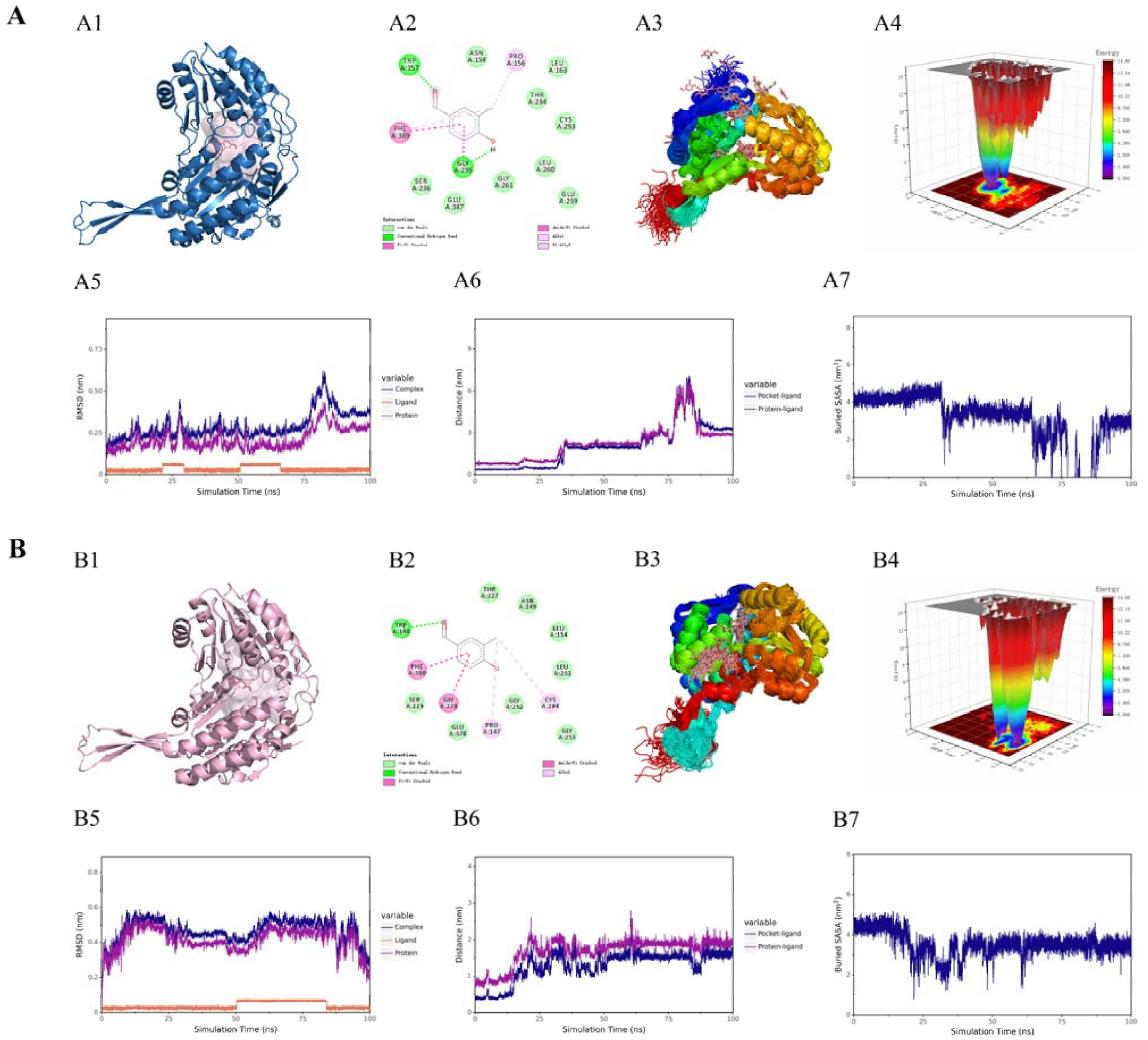

Figure S2. Docking conformations and molecular dynamics simulation results of PchA and PchA2 complexed with 4-hydroxy-3-methylbenzaldehyde (4H3MBH). (A) PchA-4H3MBH complex; (B) PchA2-4H3MBH complex. Subpanels A1/B1: 3D docking conformations of the complexes; A2/B2: 2D protein-ligand interaction diagrams; A3/B3: Structural overlays during the 100 ns simulation trajectory; A4/B4: Free energy landscapes; A5/B5: Root mean square deviation (RMSD) plots of the complex, protein, and ligand over time; A6/B6: Distance variation between the protein and the ligand-binding site; A7/B7: Buried solvent-accessible surface area (SASA) between the ligand and the protein.

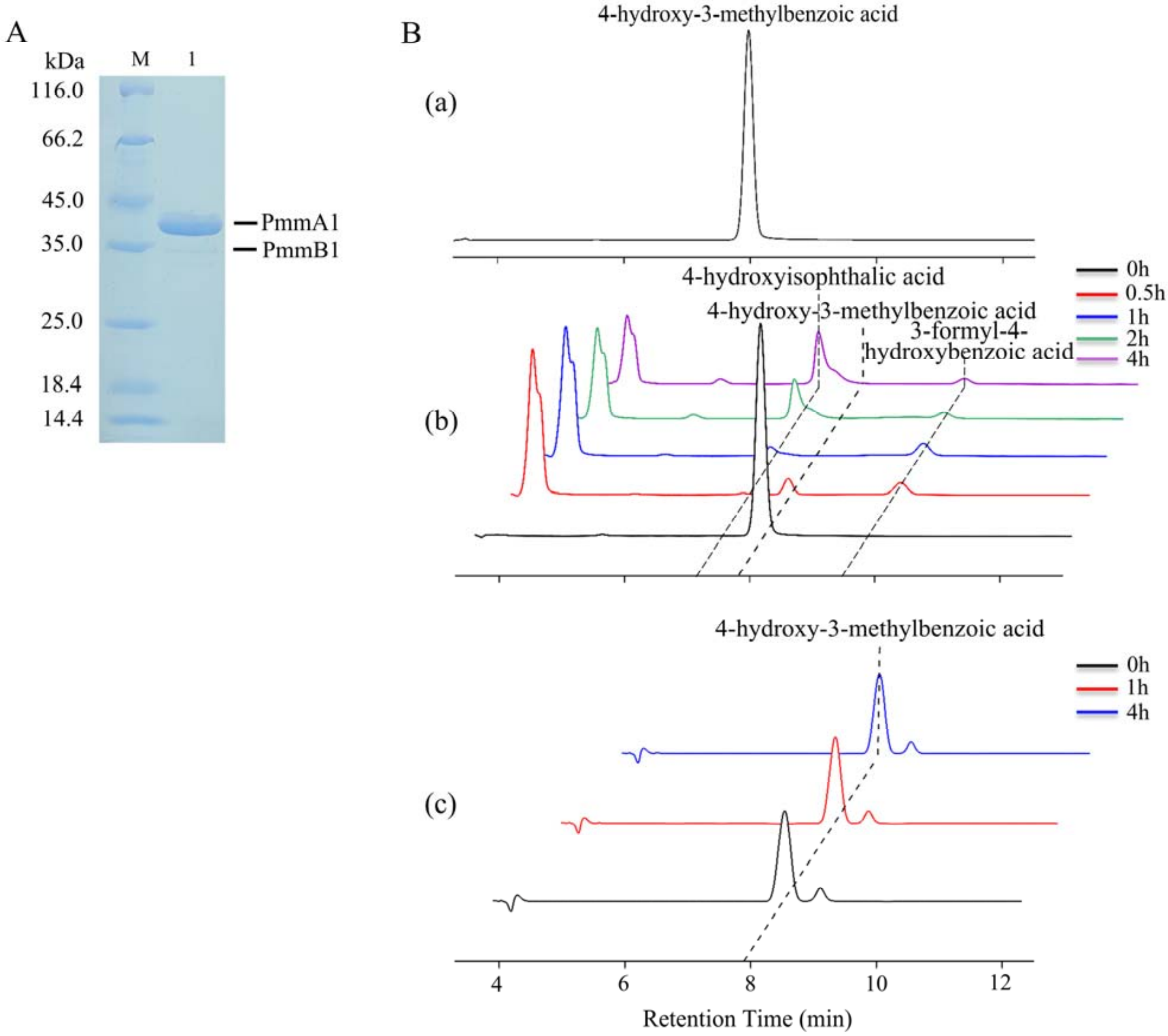

Figure S3 Purification and enzyme activity analysis of recombinant PmmA1B1. (A) SDS-PAGE analysis of purified recombinant His$_6$-PmmA1B1. The protein molecular mass standards are indicated by numbers in kDa. (B) HPLC chromatograms of biotransformation products of 4-hydroxy-3-methylbenzoic acid catalyzed by the recombinant strain *E. coli* BL21(DE3)/pET28a-*orf02368-02369* (b) and the purified recombinant His$_6$-PmmA1B1 (c). Authentic 4-hydroxy-3-methylbenzoic acid (a). Authentic 3-formyl-4-hydroxybenzoic acid and 4-hydroxyisophthalic acid are shown in Figure S6E. The strain *E. coli* BL21(DE3)/pET28a(+) was used as a negative control, and no oxidation activity towards 4-hydroxy-3-methylbenzoic acid was detected.

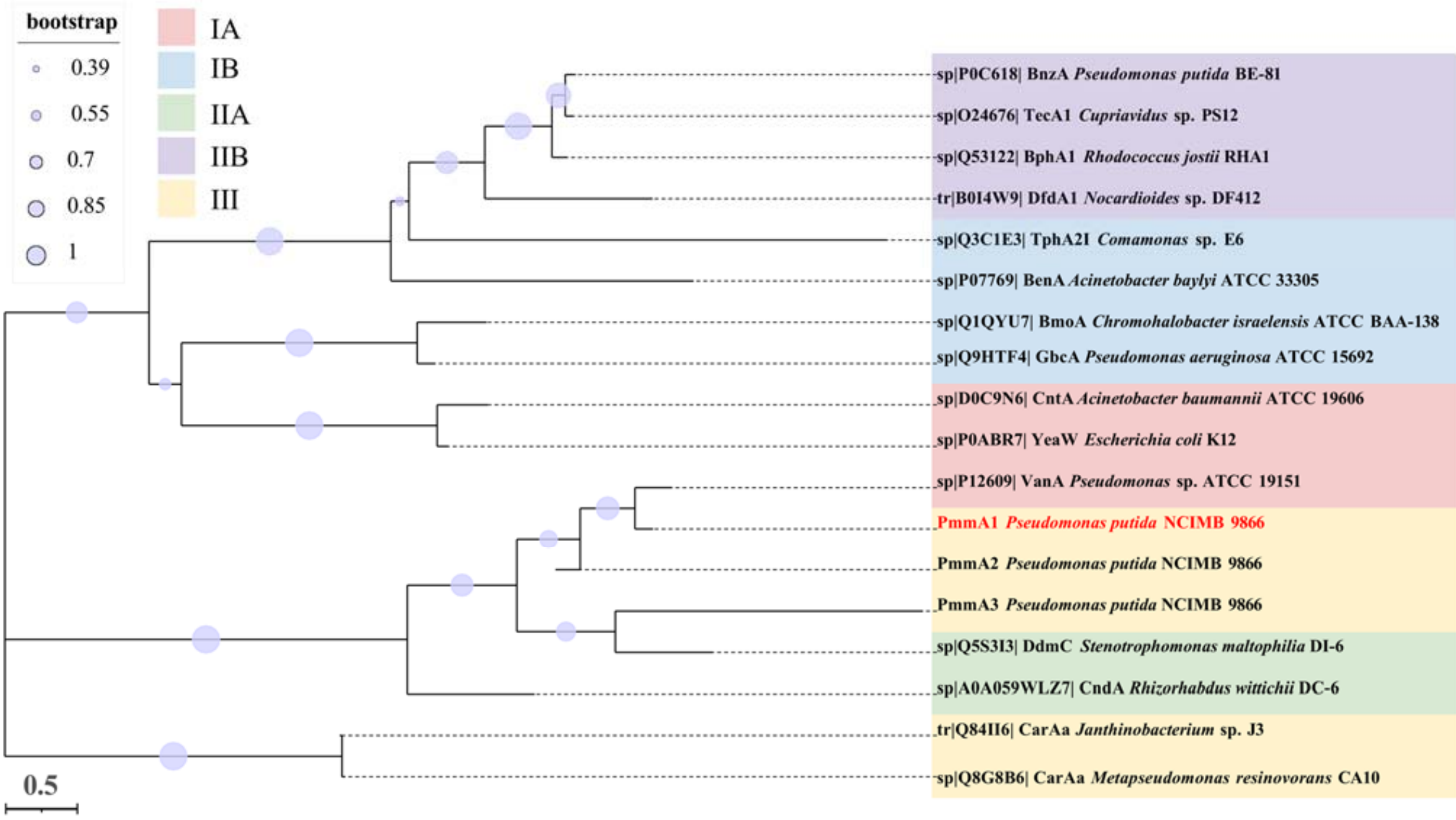

Figure S4 Phylogenetic analysis of PmmA1, PmmA2, PmmA3, and other Rieske non-heme iron-dependent oxygenase large components (oxygenase components). The tree was constructed using the neighbor-joining (NJ) method with 1000 bootstrap replications based on the amino acid sequences of the oxygenase large components. The light purple dot at each branching node represents bootstrap values. The prefix "sp" denotes the SWISS-PROT database, and "tr" denotes TrEMBL (Translation of EMBL nucleotide database), and the Uniprot ID is also indicated. The PmmA1 sequence characterized in this study is indicated in red. The subclasses IA, IB, IIA, IIB, and Class III of the Rieske non-heme iron-dependent oxygenase family are indicated by different colors.

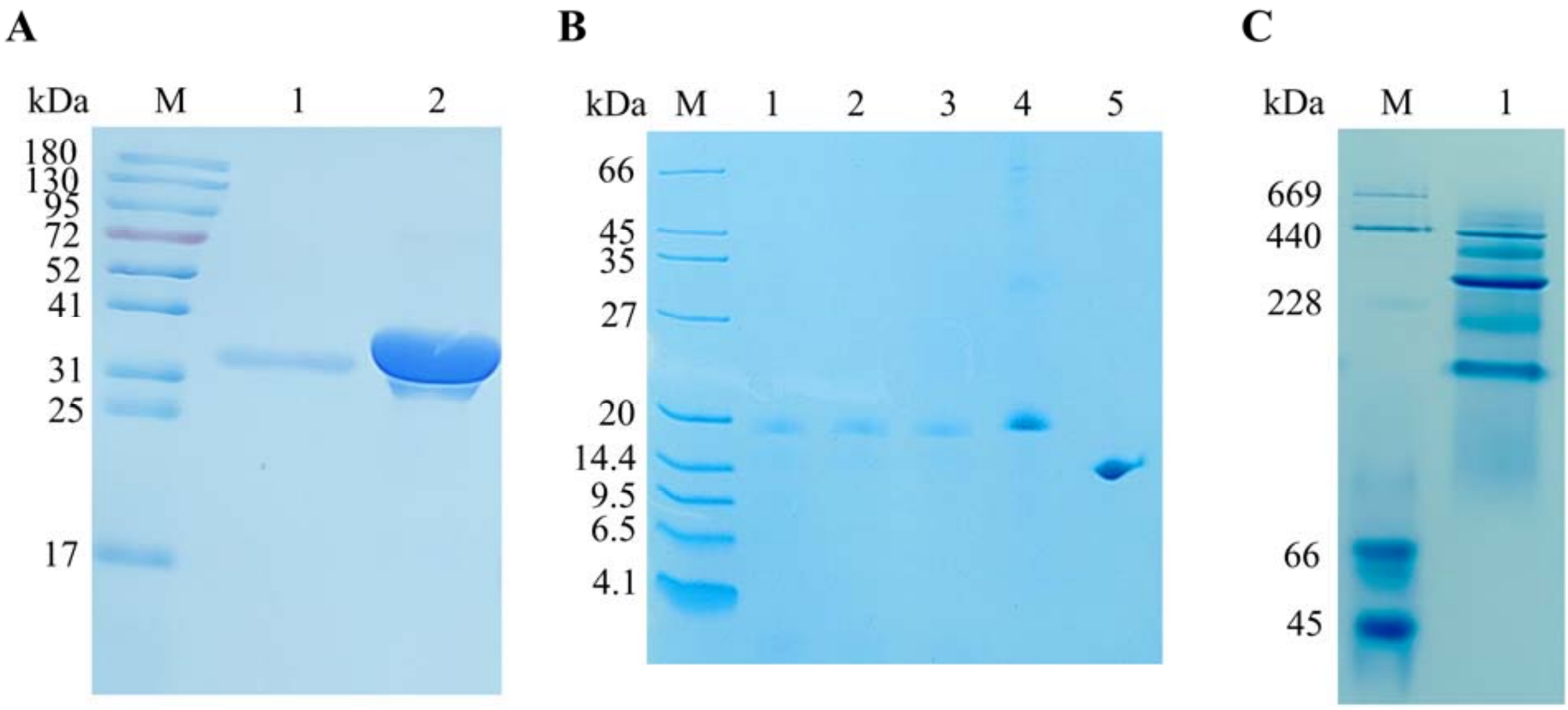

Figure S5 (A) SDS-PAGE analysis of purified recombinant His$_6$-PmmB1/Orf2368 (Lane 1) and His$_6$-PmmA1/Orf2369 (Lane 2). Lane M, RealBand tri-color pre-stained protein marker II (C610110, Sangon, Shanghai, China). (B) Tricine SDS-PAGE analysis of purified recombinant His$_6$-Orf02496 (Lane 1), His$_6$-Fdx (Lane 2), His$_6$-Orf05169 (Lane 3), His$_6$-Orf05199 (Lane 4), and His$_6$-Orf05413 (Lane 5). Lane M, non-prestained protein marker (C600201, Sangon, Shanghai, China). (C) Native-PAGE analysis of the oligomeric state of purified His$_6$-PmmA1. Lane M, high-molecular-weight non-denaturing protein marker (BL1335A, Biosharp, Beijing, China). Lane 1, Purified recombinant His$_6$-PmmA1 protein. The protein molecular mass standards are indicated by numbers in kDa.

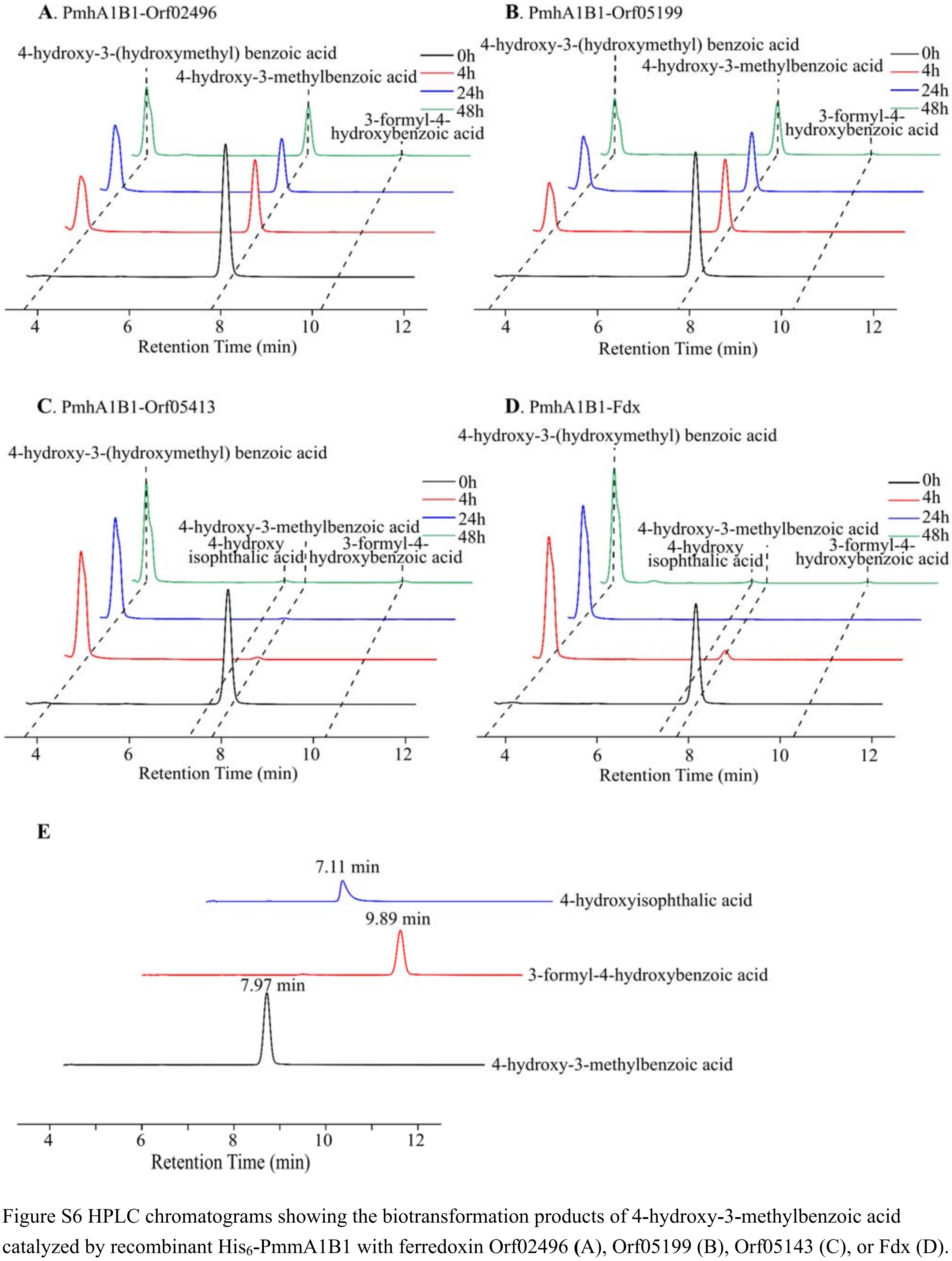

Figure S6 HPLC chromatograms showing the biotransformation products of 4-hydroxy-3-methylbenzoic acid catalyzed by recombinant His_6-PmmA1B1 with ferredoxin Orf02496 (A), Orf05199 (B), Orf05143 (C), or Fdx (D). (E) HPLC chromatograms of authentic 4-hydroxy-3-methylbenzoic acid, 3-formyl-4-hydroxybenzoic acid, and 4-hydroxyisophthalic acid.

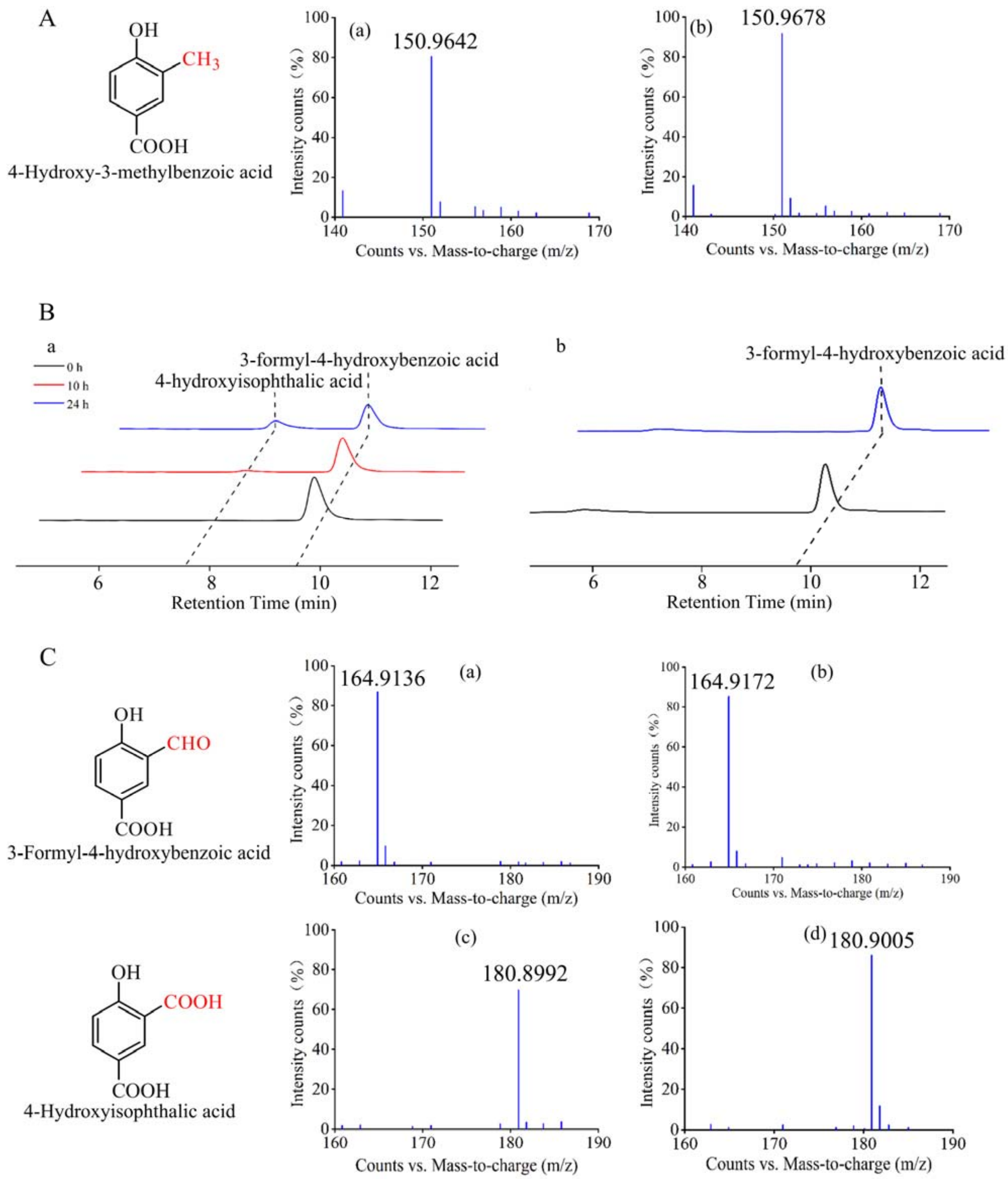

Figure S7 (A) Identification of recombinant His$_6$-PmmA1B1 and ferredoxin biotransformation products of 4-hydroxy-3-methylbenzoic acid by LC-MS(a). Authentic 4-hydroxy-3-methylbenzoic acid (b). **(**B) HPLC chromatograms of biotransformation products of 3-formyl-4-hydroxybenzoic acid catalyzed by recombinant His$_6$-PmmA1B1-Orf05169 (a) without the enzyme (b). Authentic 3-formyl-4-hydroxybenzoic acid and authentic 4-hydroxyisophthalic acid are shown in Figure S6E. (C) Identification of the recombinant His$_6$-PmmA1B1 and ferredoxin biotransformation products of 3-formyl-4-hydroxybenzoic acid by LC-MS. Mass spectra of 3-formyl-4-hydroxybenzoic acid (a) and 4-hydroxyisophthalic acid (c) captured during the biotransformation. Mass spectra of authentic 3-formyl-4-hydroxybenzoic acid (b) and authentic 4-hydroxyisophthalic acid (d).

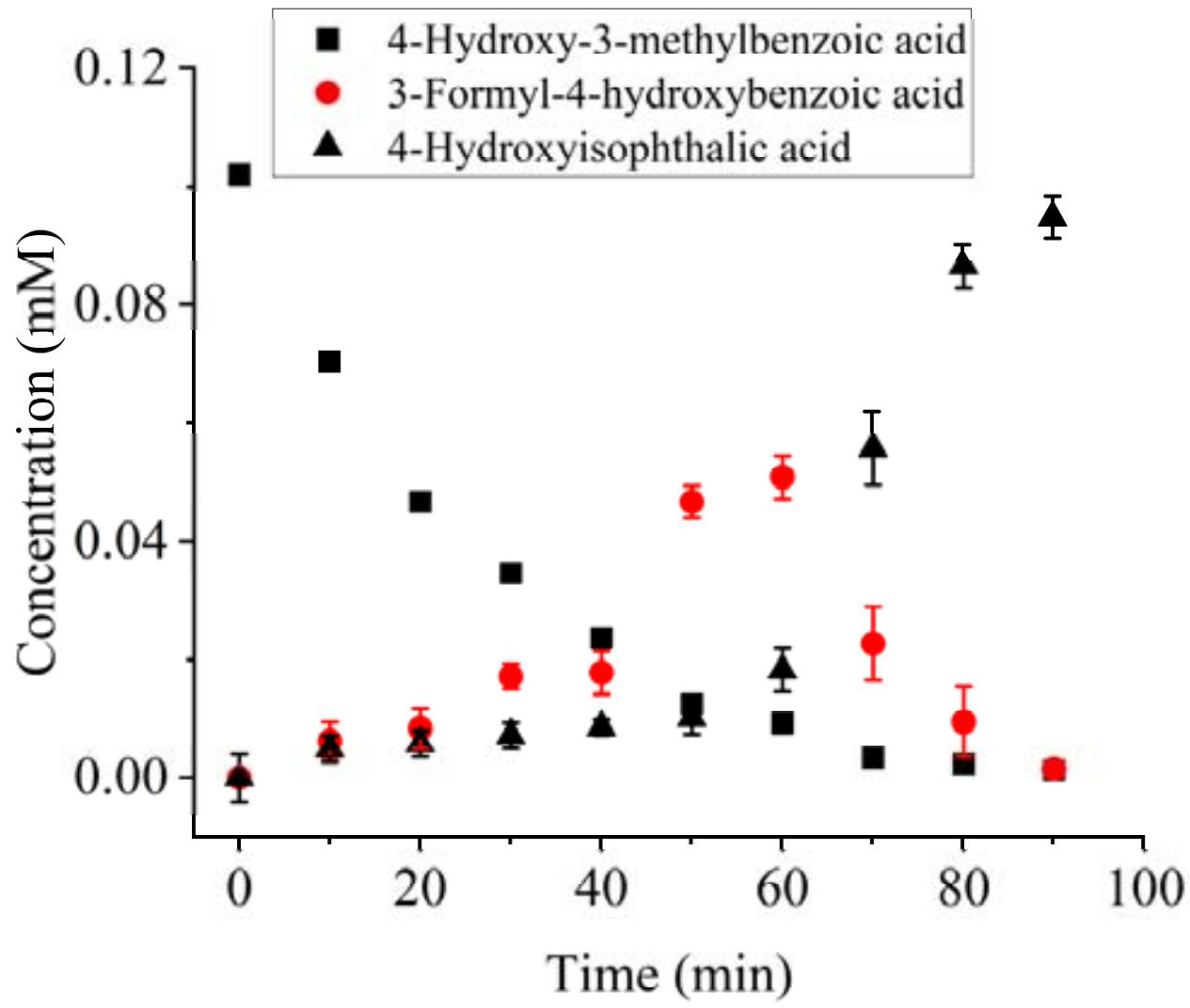

Figure S8 Time course of the oxidation of 4-hydroxy-3-methylbenzoic acid catalyzed by purified His_6-PmmA1B1-Orf05169. The consumption of 4-hydroxy-3-methylbenzoic acid, the accumulation of 3-formyl-4-hydroxybenzoic acid, and 4-hydroxyisophthalic acid were quantified via HPLC analysis. The error bars represent one SD of the mean values from three independent experiments.

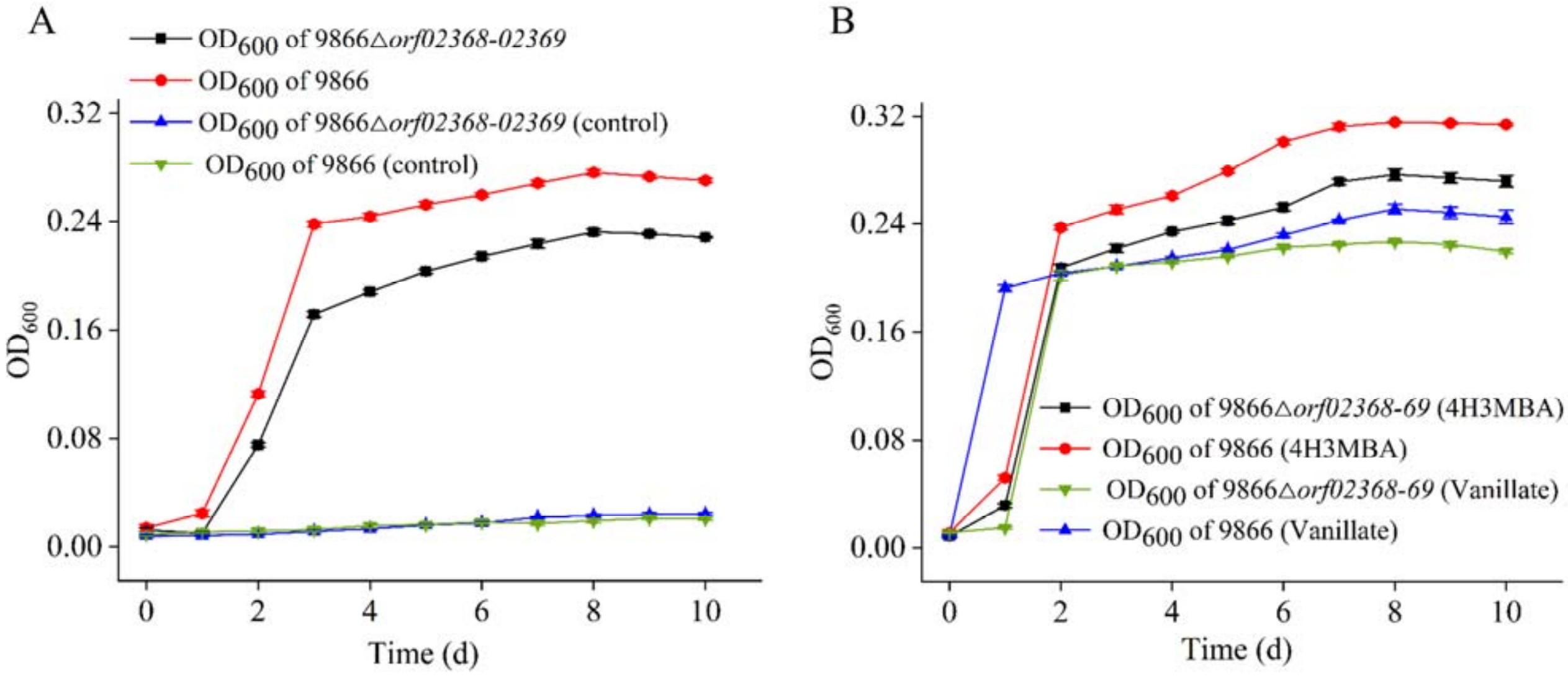

Figure S9 Growth curves of the wild-type NCIMB 9866 and the mutant strain NCIMB 9866Δ*orf02368-02369*. The strains were cultured aerobically at 30 °C in minimal medium. Growth was monitored by measuring optical density at 600 nm (OD_{600}) over time. (A) Growth curves using 2 mM 2,4-xylenol as the sole carbon source. (B) Growth curves using 2mM 4H3MBA or 2 mM vanillate as the carbon source. Error bars represent the standard deviation of three independent biological replicates.

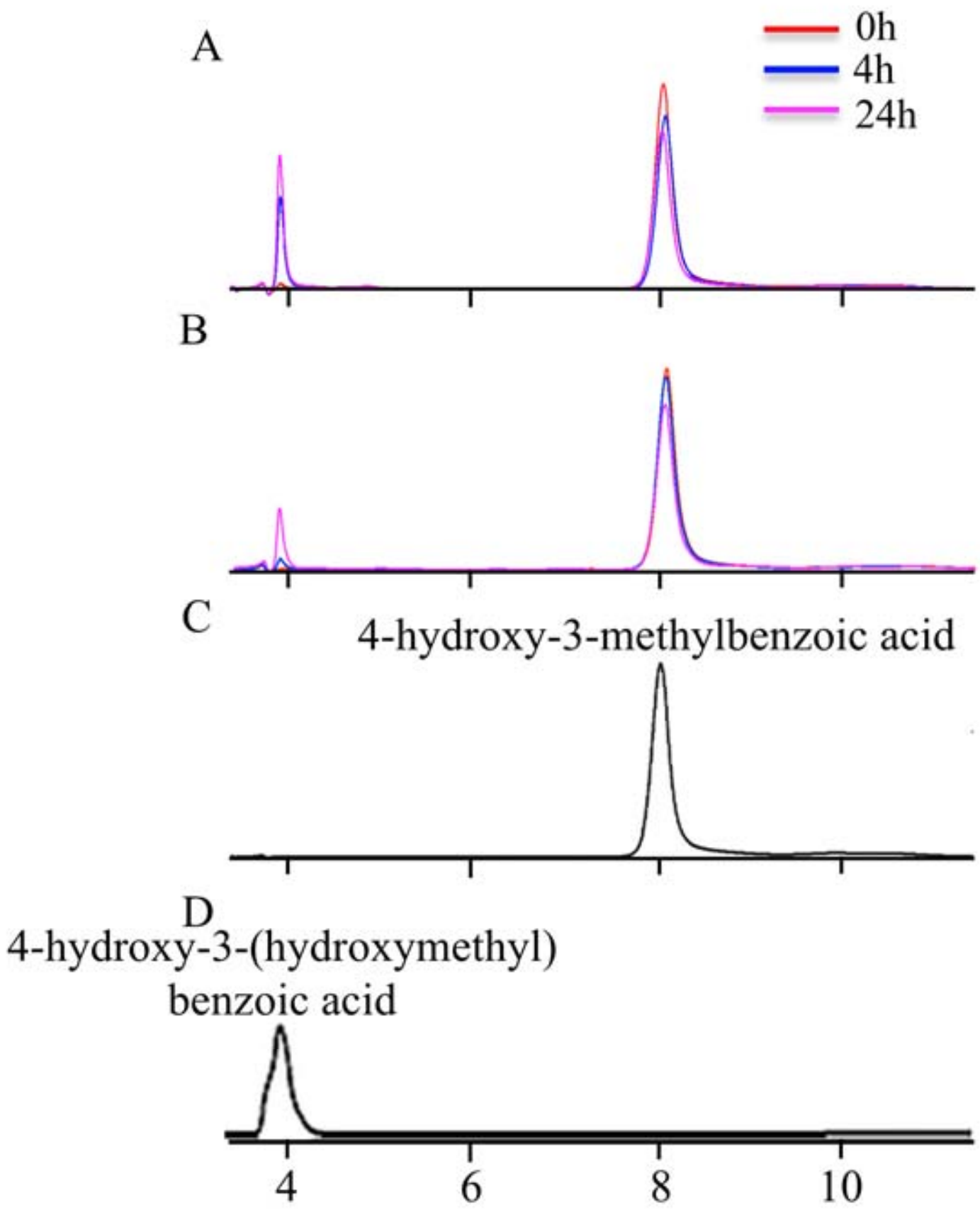

Figure S10 Enzyme activity analysis of recombinant Orf01432-01433 and Orf03582-03583 with ferredoxin Orf05169. HPLC chromatograms of biotransformation products of 4-hydroxy-3-methylbenzoic acid catalyzed by recombinant Orf01432-01433 (A) or Orf03582-03583 (B) with ferredoxin Orf05169. Authentic 4-hydroxy-3-methylbenzoic acid (C) and 4-hydroxy-3-(hydroxymethyl) benzoic acid (D).

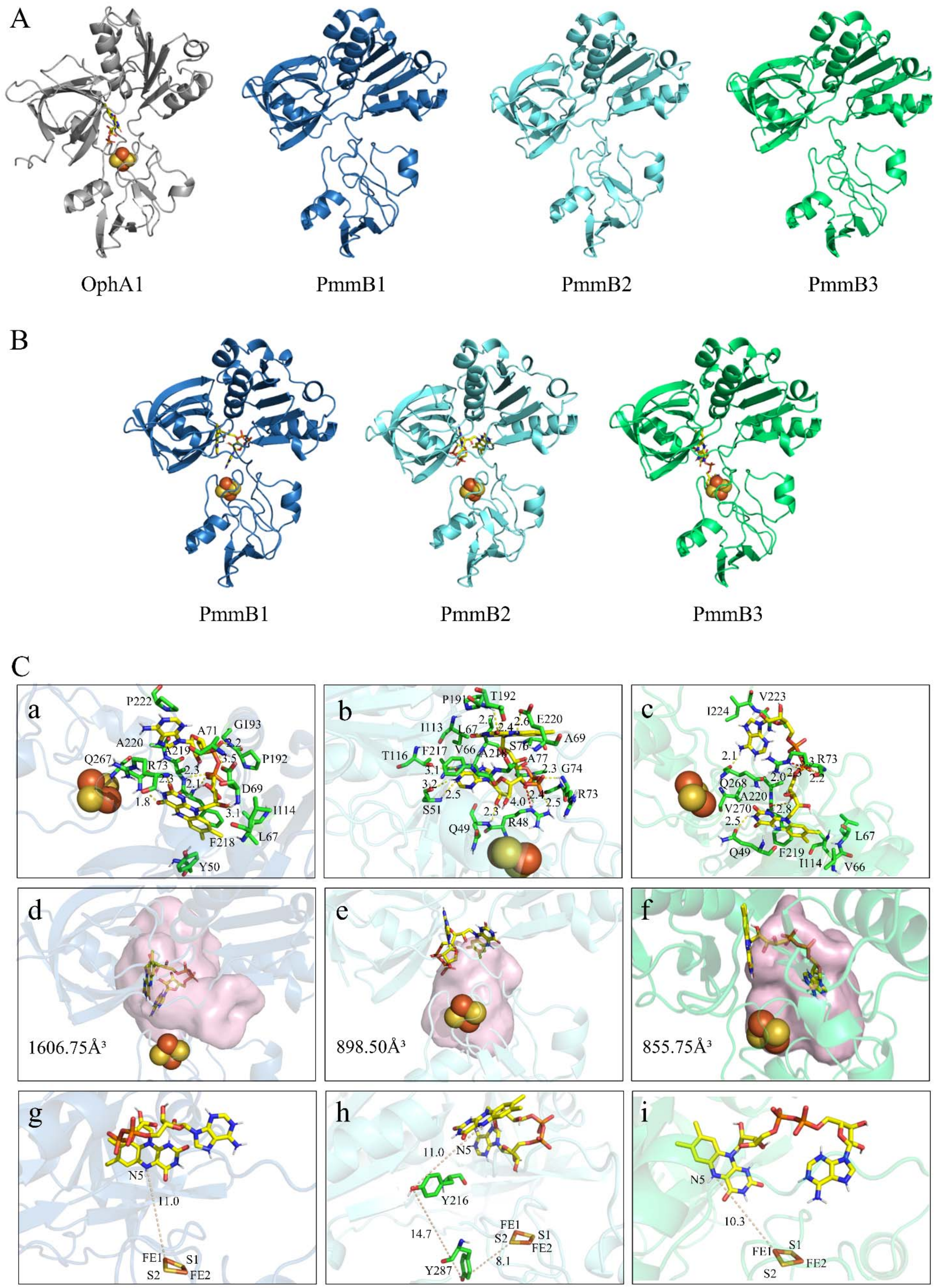

Figure S11 Analysis of the internal electron transfer pathways of the reductase components PmmB1, PmmB2, and

PmmB3. A, the structure of phthalate dioxygenase reductase OphA1 (UniProt: P33164) and the analysis of the structural models of the reductases PmmB1, PmmB2, and PmmB3 predicted using AlphaFold 3. B, a complete reductase model containing the FAD and [2Fe-2S] cofactors. FAD is represented in yellow, blue, red, and orange. The [2Fe-2S] cluster is represented in rusty orange and bright golden yellow. C, the binding patterns, binding pockets, and electron transfer pathways of the reductases PmmB1, PmmB2, and PmmB3 with FAD. a-c, molecular docking structures of PmmB1, PmmB2, and PmmB3 with FAD, the salt bridges are represented by red dotted lines, and the hydrogen bonds are represented by yellow dotted lines. d-f, the FAD binding pockets of PmmB1, PmmB2, and PmmB3. g-i, the electron transfer paths from FAD to [2Fe-2S] in PmmB1, PmmB2, and PmmB3. The electron transfer pathways, represented in wheat color, are shown with the relay residues as nodes, using the distance between the side-chain phenolic hydroxyl groups as the reference for potential tunneling jumps. Iron and sulfur are represented as orange and yellow spheres, respectively.

TABLES

Table S1 Growth characteristics of strain NCIMB 9866 and its mutants

Strain	Substrate				
	2,4-DMP	4H3MBH	4H3MBA	4HBH	4HBA
NCIMB 9866	+	+	+	+	+
NCIMB 9866Δ*pchA*	+	+	+	+	+
NCIMB 9866Δ*pchA2*	+	+	+	+	+
NCIMB 9866Δ*pchA*Δ*pchA2*	-	-	+	+	+

Note: “+”, Growth ($OD_{600} \geqslant 0.1$); “-”, No Growth.

Table S2 Specific activity of different combinations of recombinant His_6-PmmA1B1 and ferredoxin

Components	Specific activity (U/mg)[a]
His_6-PmmA1B1	0
His_6-PmmA1B1-Orf02496	0.0098 ± 0.0006
His_6-PmmA1B1-Orf05169	0.018 ± 0.001
His_6-PmmA1B1-Orf05199	0.0105 ± 0.0006
His_6-PmmA1B1-Orf05413	0.017 ± 0.001
His_6-PmmA1B1-Fdx	0.0148 ± 0.0006

[a]The error represents one SD of the mean values from the biological replicate experiments ($n \geqslant 3$).

Table S3 Substrate specificity of 4-hydroxy-3-methylbenzoate monooxygenase (His$_6$-PmmA1B1-Orf05169)

Substrate	Structure	His$_6$-PmmA1B1-Orf05169[a]
2,3-xylenol	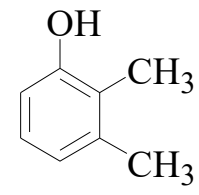	ND
2,4-xylenol	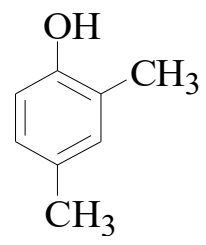	ND
2,5-xylenol	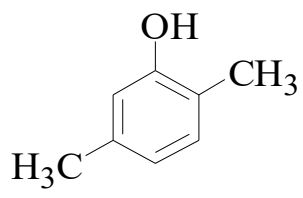	ND
2,6-xylenol	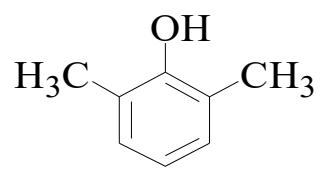	ND
3,4-xylenol	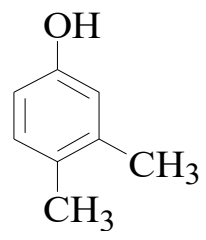	ND
3,5-xylenol	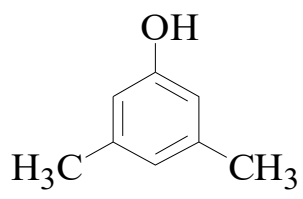	ND
m-cresol	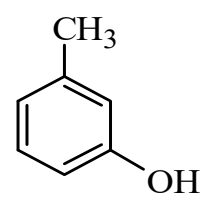	ND
o-cresol	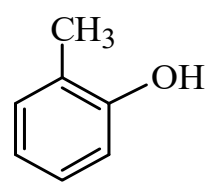	ND
p-cresol	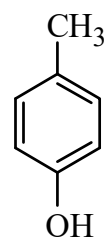	ND
Toluhydroquinone	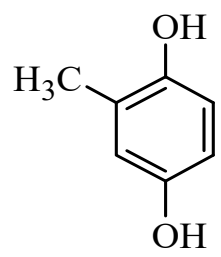	ND
2-methylterephthalate	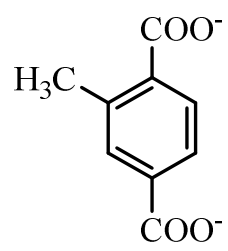	ND
2-Methoxyhydroquinone	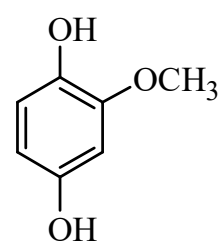	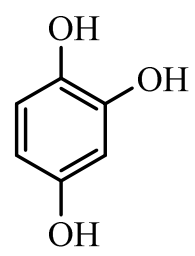
vanillate	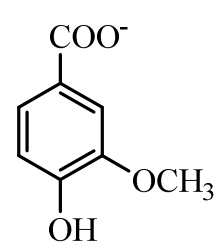	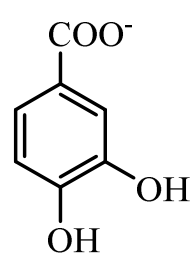

Guaiacol	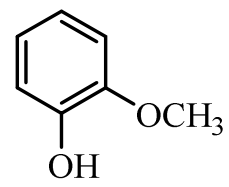	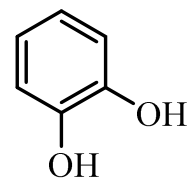
4-hydroxy-3-methylbenzoate	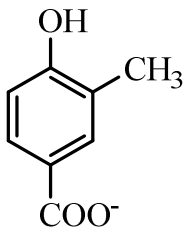	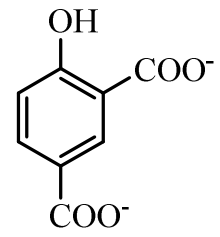
4-hydroxy-3-(hydroxymethyl) benzoate	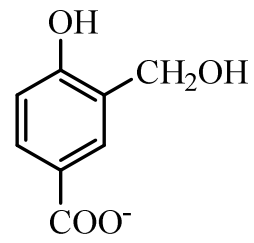	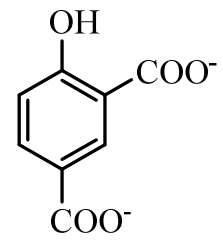
3-formyl-4-hydroxybenzoate	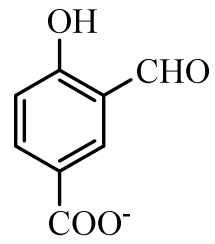	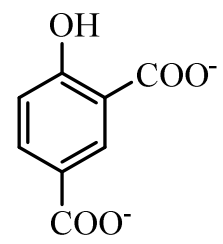

[a]The products were identified by HPLC comparison with authentic compounds. ND, the oxidative products were not detected.

Table S4 Global amino acid sequence identities of candidate oxygenase and reductase components compared to PmmA1B1 and VanAB[a]

Locus Tag	Protein name	Amino acid length (AA)	Global Identity to PmmA1 (%)	Global Identity to VanA[b] (%)	Global Identity to PmmB1 (%)	Global Identity to VanB[b] (%)
Oxygenase Components						
Orf02369	PmmA1	355	100	69.0	-	-
Orf01432	PmmA2	345	73.0	66.0	-	-
Orf03583	PmmA3	358	30.0	28.0	-	-
Orf00334	Orf00334	282	18.0	21.0	-	-
Orf03599	Orf03599	343	21.0	20.0	-	-
Orf04460	Orf04460	344	25.0	23.0	-	-
Orf05877	Orf05877	430	17.0	17.0	-	-
Reductase Components						
Orf02368	PmmB1	316	-	-	100	58.0
Orf01433	PmmB2	315	-	-	54.0	52.0
Orf03582	PmmB3	317	-	-	57.0	55.0
Orf00333	Orf00333	585	-	-	22.0	20.0
Orf03598	Orf03598	319	-	-	38.0	45.0
Orf04461	Orf04461	319	-	-	43.0	44.0
Orf05876	Orf05876	366	-	-	21.0	21.0

[a] All sequence identities were calculated using the Needleman-Wunsch global alignment algorithm (NCBI Global Align) to ensure 100% query coverage across the entire length of the respective reference proteins.

[b] VanAB is vanillate demethylase in strain *Pseudomonas* sp. ATCC 19151, and its Uniprot ID are P12609 and P12580, respectively.

"-", No data.

Table S5 Summary of the docking of PmmA1 with different substrate molecules

Protein	Molecule	binding free energy kcal/mol	Residue-Distance/Å (salt bridge)		Residue-Distance/Å (hydrogen bond)		Residue (Hydrophobic interaction)
PmmA1	4H3HMBA	-6.262	R216	3.2	W199	1.9	L154、I165、I230、V232、V254、M272、F295
					Q218	1.8	
					Q291	1.7	
					Q291	3.1	
	4H3MBA	-6.226	R216	3.2	E157	2.3	L154、I165、I230、V232 、V254、F295
					W199	1.9	
					Q218	1.8	
					Q291	1.7	
					Q291	3.1	
	3F4HBA	-6.191	R216	3.0	W199	1.9	L154、I165、I230、V232、V254、F295
					Q218	1.9	
					Q291	1.7	
					Q291	3.2	

Table S6 Main geometric parameters of the tunnels identified in the oxygenase component

Docked Complex	Tunnel	Avg_throughput	Avg_BR	Avg_L	Avg_C
PmmA1-4H3MBA	Substrate Access	0.448	0.975	14.628	1.222
	Product Release	0.117	0.809	29.337	1.796
PmmA2-4H3MBA	Substrate Access	0.451	0.989	14.930	1.251
	Product Release	0.206	0.844	23.704	1.579
PmmA3-4H3MBA	Substrate Access	0.430	0.942	16.949	1.237
	Product Release	0.332	0.757	18.279	1.766

Avg_throughput, average tunnel throughput. Avg_BR, average bottleneck radius. Avg_L, average tunnel length. Avg_C, average tunnel curvature.

Table S7 The internal electron transfer pathways and parameters of reductases

Reductase	Pocket volume (Å^3)	Distance from N5 to [2Fe-2S] (Å)	Electron transfer pathways	Total distance (Å)	Total edge weight
PmmB1	1606.75	11.0	FAD→FES	11.0	13.12
PmmB2	898.50	14.4	FAD→Y216→Y287→FES	33.8	29.59
PmmB3	855.75	10.3	FAD→FES	10.3	11.30

Table S8 Bacterial strains and plasmids used in this study

**Strain or plasmid**	**Characteristic(s) [a] or purpose**	**Reference or source**
P. putida NCIMB		
9866	Wild type strain with the phenotype of 2,4-xylenol$^+$ and *p*-cresol$^+$, Amp^R, Kan^S, Tc^S	(19)
9866Δ*pchA*	NCIMB 9866 mutant with *pchA* gene, Amp^R, Kan^R	(20)
9866Δ*pchA*/pRK415-*pchA*	*pchA* gene was complemented by pRK415-*pchA* in 9866Δ*pchA*, Amp^R, Kan^R, Tc^R	(20)
9866Δ*orf02368-02369*	NCIMB 9866 mutant with *orf02368-02369* gene, Amp^R, Kan^R	This study
9866Δ*orf02368-02369*/pRK415-*orf02368-02369*	*orf02368-02369* gene was complemented by pRK415-*orf02368-02369* in 9866Δ*orf02368-02369*, Amp^R, Kan^R, Tc^R	This study
E. coli		
Trans1-T1 Phage Resistant	F$^-$ φ80(*lacZ*)ΔM15 Δ*lac*X74 *hsdR*($r_k^-m_k^+$) Δ*recA*1398 *endA1 tonA*	TransGen Biotech
BL21(DE3)	F$^-$ *ompT hsdS*($r_B^-m_B^+$) *gal dcm*(DE3)	TransGen Biotech
WM3064	Donor strain for conjugation, 2,6-diaminopimelate auxotroph: *thrB1*004 *pro thi rpsL hsdS lacZ*ΔM15 RP4-1360 Δ(*araBAD)567* Δ*dapA*1341::[*erm pir*(wt)]	(2)
Plasmid		
pET-28a(+)	Expression vector, Kan^R, C/N-terminal His•Tag/thrombin /T7•Tag, T7 *lac* promoter, T7 transcription start, f1 origin, *lacI*	Novagen
pETDuet-1	Coexpression two target genes vector, Amp^R, His•Tag/S•Tag, T7 *lac* promoter, T7 transcription start, f1 origin, *lacI*	Novagen
pEX18Tc	Gene knockout vector, *oriT*$^+$, *sacB*$^+$, Tc^R	(21)
pRK415	Broad host range vector, Tc^R	(22)
pTn*Mod*-Okm	Kan^R, pMB1 replicon, plasposon	(23)
pET-28a-*pchA*	Expression vector for *pchA* by cloning *pchA* into the *Nde*I-*Hin*dIII restriction site of pET-28a(+) vector	(20)
pET-28a-*orf02372*	Expression vector for *pchA2* by cloning *orf02372* into the *Nde*I-*Hin*dIII restriction site of pET-28a(+) vector	This study
pET-28a-*orf00333-00334*	Expression vector for *orf00333-00334* by cloning *orf00333-00334* into the *Nde*I-*Hin*dIII restriction site of pET-28a(+) vector	This study
pET-28a-*orf01432-01433*	Expression vector for *orf01432-01433* by cloning *orf01432-01433* into the *Nde*I-*Hin*dIII restriction site of pET-28a(+) vector	This study
pET-28a-*orf02368-02369*	Expression vector for *orf02368-02369* by cloning *orf02368-02369* into the *Nde*I-*Hin*dIII restriction site of pET-28a(+) vector	This study

pET-28a-*orf03582-03583*	Expression vector for *orf03582-03583* by cloning *orf03582-03583* into the *Nde*I-*Hin*dIII restriction site of pET-28a(+) vector	This study
pET-28a-*orf03598-03599*	Expression vector for *orf03598-03599* by cloning *orf03598-03599* into the *Nde*I-*Hin*dIII restriction site of pET-28a(+) vector	This study
pET-28a-*orf04460-04461*	Expression vector for *orf04460-04461* by cloning *orf04460-04461* into the *Nde*I-*Hin*dIII restriction site of pET-28a(+) vector	This study
pETDuet-*orf02368*	Expression vector for *orf02368* by cloning *orf02368* into the *Eco*RI-*Hin*dIII restriction site of pETDuet-1 vector	This study
pETDuet-*orf02369*	Expression vector for *orf02369* by cloning *orf02369* into the *Eco*RI-*Hin*dIII restriction site of pETDuet-1 vector	This study
pETDuet-*orf02496*	Expression vector for *orf02496* by cloning *orf02496* into the *Eco*RI-*Hin*dIII restriction site of pETDuet-1 vector	This study
pETDuet-*orf05169*	Expression vector for *orf05169* by cloning *orf05169* into the *Eco*RI-*Hin*dIII restriction site of pETDuet-1 vector	This study
pETDuet-*orf05199*	Expression vector for *orf05199* by cloning *orf05199* into the *Eco*RI-*Hin*dIII restriction site of pETDuet-1 vector	This study
pETDuet-*orf05413*	Expression vector for *orf05413* by cloning *orf05413* into the *Eco*RI-*Hin*dIII restriction site of pETDuet-1 vector	This study
pETDuet-*fdx*	Expression vector for *fdx* by cloning *fdx* into the *Eco*RI-*Hin*dIII restriction site of pETDuet-1 vector	This study
pEX18Tc-*orf02368-02369*	*orf02368-02369* gene knockout vector containing two DNA fragments homologous to the upstream and downstream regions of the *orf02368-02369* and kanamycin resistance gene from pTn*Mod*-Okm in pEX18Tc vector	This study
pRK415- *orf02368-02369*	*orf02368-02369* gene complementation vector by fusing *orf02368-02369* into the *Hin*d III-*Eco*R I restriction site of pRK415 vector	This study

[a]2,4-xylenol$^+$, *p*-cresol$^+$, the ability to grow on 2,4-xylenol and *p*-cresol, respectively; Amp^R, Kan^R, Tc^R, resistant to ampicillin, kanamycin, and tetracycline, respectively; Kan^S, Tc^S, sensitive to kanamycin and tetracycline, respectively.

Table S9 Primers used in this study

Primer	Sequences (5'→3')	Purpose
orf00333-F1	gccgcgaaaccagttcatgc	Transcription analysis of *pmmB4*
orf00333-R1	aggagacatgttgctcgcctc	
orf01433-F1	ccagccttcgaagtcaccattgc	Transcription analysis of *pmmB2*
orf01433-R1	atccggagtgccctccagaac	
orf02368-F2	aactggcctgcaacggcgac	Transcription analysis of *pmmB1*
orf02368-R2	tgcccagcacttgggcgatg	
orf03582-F1	cctggccatggcgtatgaac	Transcription analysis of *pmmB3*
orf03582-R1	gctgttgtggaccgttgtcatcg	
orf03598-F1	tgacggcgcgttttgccgag	Transcription analysis of *pmmB5*
orf03598-R1	gccgccatgatcgtgcaacc	
orf04461-F1	ttcgacgctgaccgtcctgc	Transcription analysis of *pmmB6*
orf04461-R1	gttcggtatcgcgggtgacg	
orf05877-F1	gatgccaccaacgaccaggatc	Transcription analysis of *pmmB7*
orf05877-R1	cggctctgcaccaaggttctc	
RT-16S rRNA-F	ttgacgttaccgacagaataagc	Transcription analysis of 16S rRNA
RT-16S rRNA-R	gatgcagttcccaggttgagc	
pET28a-2372E01	ccgcgcggcagccatttgcaggtgcctttgctgattgg	Expression of *orf02372*
pET28a-2372E02	tcgagtgcggccgcactagatggggtaatgcctggggcc	
pET28a-333-34E01	ccgcgcggcagccatcacgtagaaaacagctttgtaatcgacg	Expression of *orf00333-00334*
pET28a-333-34E02	tcgagtgcggccgcattaaatattcaacaccagcttcttgg	
pET28a-1432-33E01	ccgcgcggcagccatttccaacggaatgcgtggtatgtc	Expression of *orf01432-01433*
pET28a-1432-33E02	tcgagtgcggccgcattagagatcaaggaccaaactattgctg	
pET28a-2368-69E01	ccgcgcggcagccattacccgaaaaacacctggtacgtcg	Expression of *orf02368-02369*
pET28a-2368-69E02	tcgagtgcggccgcattacaggtccagtaccagcagg	
pET28a-3582-83E01	ccgcgcggcagccatcttcaagtaaacgttagccgc	Expression of *orf03582-03583*
pET28a-3582-83E02	tcgagtgcggccgcatcaactaacggagttgcaaag	
pET28a-3598-99E01	ccgcgcggcagccatacgactcaagcaacccagcg	Expression of *orf03598-03599*
pET28a-3598-99E02	tcgagtgcggccgcactacaggtcgagcgtgagggtc	
pET28a-4460-61E01	ccgcgcggcagccataacatgcatgtaaccgtcgacc	Expression of *orf04460-04461*
pET28a-4460-61E02	tcgagtgcggccgcatcacagatcaagcaccaaccgc	
9866-2368-69m01	tcgagctcggtaccccgcggcaacgcttgaagccg	*orf02368-02369* knockout
9866-2368-69m02	gaaggacctccaattgttgttg	
9866-2368-69m03	aattggaggtccttcaacgcctcaggacgggggc	
9866-2368-69m04	ctctagaggatcccctgggggtcatgcccaggtgc	
orf02368-02369E3	gacggccagtgaattatgtacccgaaaaacacctggtacgtcg	Complementation of *orf02368-69* gene
orf02368-02369E4	tgattacgccaagctttacaggtccagtaccagcagg	

Table S10 Cofactor content of purified protein components

Protein Component	Iron (mol/mol protein)[a]	Sulfur (mol/mol protein)[a]	Flavin (mol/mol protein)[a]
PmmA1 (Oxygenase)	2.6 ± 0.3	1.9 ± 0.3	N/A
PmmB1 (Reductase)	1.8 ± 0.1	1.7 ± 0.2	0.86 ± 0.05
Orf05169 (Ferredoxin)	2.0 ± 0.4	1.9 ± 0.2	N/A

[a]The error represents one SD of the mean values from the biological replicate experiments ($n \geqslant 3$).

REFERENCES

1. Green MR, Sambrook J. 2012. Molecular cloning: a laboratory manual, 4th ed., 3 ed. Cold Spring Harbor Laboratory Press, New York.
2. Saltikov CW, Newman DK. 2003. Genetic identification of a respiratory arsenate reductase. Proc Natl Acad Sci USA 100:10983–10988. https://doi.org/10.1073/pnas.1834303100
3. Schägger H. 2006. Tricine-SDS-PAGE. Nat Protoc 1:16–22. https://doi.org/10.1038/nprot.2006.4
4. Carter P. 1971. Spectrophotometric determination of serum iron at the submicrogram level with a new reagent (ferrozine). Anal Biochem 40:450–458. https://doi.org/10.1016/0003-2697(71)90405-2
5. Stookey LL. 1970. Ferrozine---a new spectrophotometric reagent for iron. Anal Chem 42:779–781. https://doi.org/10.1021/ac60289a016
6. Beinert H. 1983. Semi-micro methods for analysis of labile sulfide and of labile sulfide plus sulfane sulfur in unusually stable iron-sulfur proteins. Anal Biochem 131:373–378. https://doi.org/10.1016/0003-2697(83)90186-0
7. Ghisla S, Massey V, Lhoste J-M, Mayhew SG. 1974. Fluorescence and optical characteristics of reduced flavines and flavoproteins. Biochemistry-US 13:589–597. https://doi.org/10.1021/bi00700a029
8. Ghisla S. 1980. [51] Fluorescence and optical characteristics of reduced flavins and flavoproteins, p 360–373, Methods in Enzymology, vol 66. Academic Press.
9. Sporty JL, Kabir MM, Turteltaub KW, Ognibene T, Lin S-J, Bench G. 2008. Single sample extraction protocol for the quantification of NAD and NADH redox states in *Saccharomyces cerevisiae*. J Sep Sci 31:3202–3211. https://doi.org/10.1002/jssc.200800238
10. Abraham M, Murtola T, Schulz R, Páll S, Smith J, Hess B, Lindahl E. 2015. GROMACS: High performance molecular simulations through multi-level parallelism from laptops to supercomputers. SoftwareX 1-2:19–25. https://doi.org/10.1016/j.softx.2015.06.001
11. Maier JA, Martinez C, Kasavajhala K, Wickstrom L, Hauser KE, Simmerling C. 2015. ff14SB: Improving the Accuracy of Protein Side Chain and Backbone Parameters from ff99SB. J Chem Theory Comput 11:3696–3713. https://doi.org/10.1021/acs.jctc.5b00255
12. Wang J, Wang W, Kollman P, Case D. 2006. Automatic atom type and bond type perception in molecular mechanical calculations. J Mol Graphics Modell 25:247–260. https://doi.org/10.1016/j.jmgm.2005.12.005
13. Valdés-Tresanco MS, Valdés-Tresanco ME, Valiente PA, Moreno E. 2021. gmx_MMPBSA: A New Tool to Perform End-State Free Energy Calculations with GROMACS. J Chem Theory Comput 17:6281–6291. https://doi.org/10.1021/acs.jctc.1c00645
14. Chovancova E, Pavelka A, Benes P, Strnad O, Brezovsky J, Kozlikova B, Gora A, Sustr V, Klvana M, Medek P, Biedermannova L, Sochor J, Damborsky J. 2012. CAVER 3.0: A Tool for the Analysis of Transport Pathways in Dynamic Protein Structures. PLoS Comput Biol 8:e1002708. https://doi.org/10.1371/journal.pcbi.1002708
15. Zdobnov EM, Apweiler R. 2001. InterProScan – an integration platform for the signature-recognition methods in InterPro. Bioinformatics 17:847–848. https://doi.org/10.1093/bioinformatics/17.9.847
16. Hekkelman ML, de Vries I, Joosten RP, Perrakis A. 2023. AlphaFill: enriching AlphaFold models with ligands and cofactors. Nat Methods 20:205–213. https://doi.org/10.1038/s41592-022-01685-y
17. Xu Y, Wang S, Hu Q, Gao S, Ma X, Zhang W, Shen Y, Chen F, Lai L, Pei J. 2018. CavityPlus: a web server for protein cavity detection with pharmacophore modelling, allosteric site identification and covalent ligand binding ability prediction. Nucleic Acids Res 46:W374–W379. https://doi.org/10.1093/nar/gky380
18. Tazhigulov RN, Gayvert JR, Wei M, Bravaya KB. 2019. eMap: A Web Application for Identifying and Visualizing

Electron or Hole Hopping Pathways in Proteins. J Phys Chem B 123:6946–6951. https://doi.org/10.1021/acs.jpcb.9b04816

19. Chapman PJ, Hopper DJ. 1968. The bacterial metabolism of 2,4-xylenol. Biochem J 110:491–8. https://doi.org/10.1042/bj1100491

20. Chen YF, Chao H, Zhou NY. 2014. The catabolism of 2,4-xylenol and *p*-cresol share the enzymes for the oxidation of *para*-methyl group in *Pseudomonas putida* NCIMB 9866. Appl Microbiol Biotechnol 98:1349–56. https://doi.org/10.1007/s00253-013-5001-z

21. Hoang TT, Karkhoff-Schweizer RR, Kutchma AJ, Schweizer HP. 1998. A broad-host-range Flp-FRT recombination system for site-specific excision of chromosomally-located DNA sequences: application for isolation of unmarked *Pseudomonas aeruginosa* mutants. Gene 212:77–86. https://doi.org/10.1016/s0378-1119(98)00130-9

22. Keen NT, Tamaki S, Kobayashi D, Trollinger D. 1988. Improved broad-host-range plasmids for DNA cloning in gram-negative bacteria. Gene 70:191–7. https://doi.org/10.1016/0378-1119(88)90117-5

23. Dennis JJ, Zylstra GJ. 1998. Plasposons: Modular self-cloning minitransposon derivatives for rapid genetic analysis of gram-negative bacterial genomes. Appl Environ Microbiol 64:2710–2715. https://doi.org/10.1128/AEM.64.7.2710-2715.1998
